# Genome-wide association study reveals 18 QTL for major agronomic traits in a Nordic–Baltic spring wheat germplasm

**DOI:** 10.3389/fpls.2024.1393170

**Published:** 2024-06-21

**Authors:** Andrius Aleliūnas, Andrii Gorash, Rita Armonienė, Ilmar Tamm, Anne Ingver, Māra Bleidere, Valentīna Fetere, Hannes Kollist, Tomasz Mroz, Morten Lillemo, Gintaras Brazauskas

**Affiliations:** ^1^ Institute of Agriculture, Lithuanian Research Centre for Agriculture and Forestry, Akademija, Lithuania; ^2^ Centre of Estonian Rural Research and Knowledge, Jõgeva Alevik, Estonia; ^3^ Crop Research Department, Institute of Agricultural Resources and Economics, Stende Research Centre, Dižstende, Latvia; ^4^ Institute of Bioengineering, University of Tartu, Tartu, Estonia; ^5^ Department of Plant Sciences, Norwegian University of Life Sciences, Ås, Norway

**Keywords:** GWAS, yield-related traits, QTL, Nordic region, *Triticum aestivum*

## Abstract

Spring wheat (*Triticum aestivum* L.) remains an important alternative to winter wheat cultivation at Northern latitudes due to high risk of overwintering or delayed sowing of winter wheat. We studied nine major agronomic traits in a set of 299 spring wheat genotypes in trials across 12-year-site combinations in Lithuania, Latvia, Estonia, and Norway for three consecutive years. The dataset analyzed here consisted of previously published phenotypic data collected in 2021 and 2022, supplemented with additional phenotypic data from the 2023 field season collected in this study. We combined these phenotypic datasets with previously published genotypic data generated using a 25K single nucleotide polymorphism (SNP) array that yielded 18,467 markers with a minor allele frequency above 0.05. Analysis of these datasets via genome-wide association study revealed 18 consistent quantitative trait loci (QTL) replicated in two or more trials that explained more than 5% of phenotypic variance for plant height, grain protein content, thousand kernel weight, or heading date. The most consistent markers across the tested environments were detected for plant height, thousand kernel weight, and days to heading in eight, five, and six trials, respectively. No beneficial effect of the semi-dwarfing alleles *Rht-B1b* and *Rht-D1b* on grain yield performance was observed across the 12 tested trials. Moreover, the cultivars carrying these alleles were low yielding in general. Based on principal component analysis, wheat genotypes developed in the Northern European region clustered separately from those developed at the southern latitudes, and markers associated with the clustering were identified. Important phenotypic traits, such as grain yield, days to heading, grain protein content, and thousand kernel weight were associated with this clustering of the genotype sets. Interestingly, despite being adapted to the Nordic environment, genotypes in the Northern set demonstrated lower grain yield performance across all tested environments. The results indicate that spring wheat germplasm harbors valuable QTL/alleles, and the identified trait-marker associations might be useful in improving Nordic–Baltic spring wheat germplasm under global warming conditions.

## 1 Introduction

Bread wheat (*Triticum aestivum* L.) is an important source of food contributing approximately 20% of the total dietary calories and proteins consumed by humans (Shewry and Hey, 2015). Sustaining a continual increase in wheat yields is a key component to address the challenge of rising food demand. However, climate change threatens global wheat production and productivity. In response to this threat, prioritizing the adaptation of wheat varieties to future climates within breeding strategies is imperative. The study by Xiong et al. (2021) provides evidence that climate change affects wheat breeding and may increase genotype-environment interactions. However, the implementation of stress tolerance breeding strategies is aiding wheat’s adaptation to current, warmer climates, thereby creating opportunities for greater and faster genetic gains under rising temperatures. Improved cultivars with maximized yield potential are required to reduce the gap between supply and demand (Tian et al., 2021).

Europe is one of the largest wheat producers globally, comprising 33% of the total world wheat production (FAO, 2021). Grain yield (GY) production of wheat is predicted to shift to Northern latitudes due to increasing temperatures and precipitation during the winter–spring season. Indeed, the highest growth rates in wheat yield were observed in Latvia (92%), followed by Estonia (87%), and Lithuania (86%) from 1993–1997 to 2013–2017 (Pinke et al., 2022). However, increasing summer temperatures have negatively impacted wheat yields in Northern Europe, with decreases of 450 kg ha^−1^ per 1°C increase, despite the positive effects of overall rainfall, winter rainfall, and winter temperatures (Lopes, 2022). Higher summer temperatures are shortening the grain filling period, thus limiting GYs (Schittenhelm et al., 2020). Furthermore, regularly observed late spring droughts in Northern Europe are particularly damaging for spring wheat because, unlike winter wheat, roots in spring wheat tend to be shorter at that time thus restricting access to water from the deeper soil layers (Chawade et al., 2018).

The total GY depends on multiple yield components, where each component is inherited in a quantitative manner, that is, they are controlled by numerous, small-effect quantitative trait loci (QTL) (Zhang et al., 2010; Würschum et al., 2017). Genome-wide association study (GWAS), based on linkage disequilibrium (LD), is a powerful tool to explore complex QTL and allelic variants that are associated with phenotypic variation (Alqudah et al., 2020). GWAS has been successfully applied to identify QTL and candidate genes for GY and yield-related traits in bread wheat (Gahlaut et al., 2019; Alqudah et al., 2020; Lin et al., 2021; Said et al., 2022).

The countries of the Baltic and Nordic regions differ in duration of the growing season due to differences in their geographic positions. In regions at northern latitudes, the challenges posed by wet periods toward the end of the growing season, including lodging and quality degradation due to pre-harvest sprouting, often lead to a preference for early maturing varieties of spring cereals. Early maturity played a pivotal role in the breeding of Nordic spring wheat, facilitating the expansion of the spring wheat area in Sweden during the first half of the 20th century. The landraces of Nordic spring wheat have been shown to be heading and maturing earlier than Nordic cultivars (Diederichsen et al., 2013). However, in response to the effect of climate change, there has been an extension of the vegetative season in Nordic countries. As evidenced by a study of Mróz et al. (2022), varieties introduced after 1970 have demonstrated adaptation to this shift by extending their vegetative periods by an average of four days. This enabled the development of higher yielding cultivars with high-protein content characterized by delayed maturation, extended grain-filling periods, and augmented grain production per spike and per unit area (Mróz et al., 2022).

The incorporation of “Green revolution” semi-dwarfing alleles (*Rht-B1b* and *Rht-D1b*) into new cultivars had a pronounced effect on the global wheat yield increase in the 1960s (Wang et al., 2014). New semi-dwarf wheat cultivars had thicker and shorter stems, enabling them to carry heavy, fully filled spikes without lodging resulting in superior GYs compared to wild-type-old cultivars. However, semi-dwarf cultivars need to meet specific environmental conditions to manifest their superiority. Yield improvement can be achieved under well-watered conditions and with the application of high levels of nitrogen fertilizers. The *Rht-B1b* and *Rht-D1b* alleles lead to shorter coleoptile length, weaker early vigor, and higher susceptibility to drought stress that result in smaller thousand grain weight and reduced yield, indicating their potential weakness in adaptation under a global warming scenario (Jatayev et al., 2020; Tian et al., 2022). Liu et al. (2022) demonstrated that cultivars possessing semi-dwarfing alleles experience approximately a 25% higher yield penalty in conditions of increased temperature compared to cultivars with wild-type alleles. If the temperature increases by 1°C, genotypes possessing *Rht-D1b* may lose approximately 23.5% of the yield. Therefore, the Green Revolution genes should be replaced with other *Rht* genes, such as *Rht8* and *Rht24*, which do not induce vulnerability under drought conditions, to ensure food security in a global warming scenario (Würschum et al., 2017; Tian et al., 2022; Xu et al., 2023).

In the present study, a total of 299 spring wheat genotypes were evaluated in multiple trials to improve our understanding of the genetic basis of GY and GY-related traits. A published phenotypic data set collected in 2021 and 2022 (Ingver et al., 2023, Preprint) was supplemented with additional data from the 2023 field season and previously published genotypic data to undertake GWAS. Here, the identification of replicated QTL for key agronomic traits represents a valuable resource for the introduction into spring wheat breeding programs, especially in the Nordic and Baltic region.

## 2 Materials and methods

### 2.1 Plant material and field trials

The NOBAL wheat collection, consisting of 299 spring wheat cultivars and breeding lines sourced from the Baltic countries (Estonia, Latvia, and Lithuania) (151 accessions), Nordic countries (64 accessions), Central and Western Europe (74 accessions), and 10 exotic genotypes (**Supplementary Table S1**) was studied at four locations (Lithuania, Latvia, Estonia, and Norway) over three consecutive growing seasons from 2021 to 2023.

Field trials were carried out at the Lithuanian Research Centre for Agriculture and Forestry (Dotnuva, in central Lithuania, 55.39°N, 23.88°E), Stende Research Centre (Dižstende, north-western Latvia, 57.18°N, 22.56°E), the Centre of Estonian Rural Research and Knowledge (Jõgeva, Eastern part of Estonia, 58.76°N, 26.24°E) and the Vollebekk Research Station of the Norwegian University of Life Sciences (Ås, south-eastern Norway, 59.65°N, 10.75°E). The design of the trials and applied management practices were described earlier (Ingver et al., 2023 Preprint). In short, the genotypes were sown in alpha-lattice design with two replications. Conventional tillage and recommended local crop management practices were followed. Herbicides were selected for the prevalent weed species and foliar fungicides were applied to prevent fungal diseases. Insecticides were used when the insect populations reached an economic threshold. The same crop management practice was also followed during the field season of 2023.

### 2.2 Measured traits

The collection previously phenotyped by (Ingver et al., 2023, Preprint) for the seven agronomic traits, such as days to heading (DH), days to maturity (MAT), plant height (PH), thousand-kernel weight (TKW), grain protein content (GPC), grain test weight (TW), and GY was supplemented with phenotypic data collected during 2023 field season (**Supplementary Table S2**). Agronomic traits were measured following the procedures described by (Ingver et al., 2023 Preprint). Additionally, DH were converted into growing degree days (GDDs) and photo thermal units (PTUs) to account for temperature and daylight variability from sowing to heading at each environment, respectively.

GDDs were calculated using the Equation 1:


(1) $$\frac{\left(Tmax+Tmin\right)}{2}-Tbase$$

Where, T_max_ is daily highest temperature and T_min_ is daily lowest temperature, T_base_ is 5°C. PTU was calculated by multiplying GDD by photoperiod length at each trial location. The photoperiod length at the trial sites was extracted using a *geosphere* package (Hijmans, 2022) for R.

GPC and TW were measured using near-infrared reflectance (NIR) spectrometer (Perten Inframatic 9200/Perten Instrument AB) in Norway, and near-infrared transmittance (NIT) whole grain analyzers were used in Estonia (InfratecTM/Foss), Latvia (InfratecTM NOVA/Foss), and Lithuania (Infratec 1241/Foss).

A detailed description of the phenotypic measurements can be found in **Table 1**.

**Table 1:** Agronomic traits included in analysis.

Trait	Abbreviation	Method of measurements	Unit/scale
Grain yield	GY	Measured at 14% moisture	g m^−2^
Days to heading	DH	The number of days from sowing to heading. The heading date was recorded when more than 70% of the plants showed 70% spike emergence from the flag leaf sheath.	days
Days to maturity	MAT	The number of days from sowing until physiological maturity.	days
Growing degree days	GDD	GDD = (T_max_ + T_min_)/2 – T_base_	heat units
Photo thermal units	PTU	GDD × day length	photo thermal units
Plant height	PH	Measured in three different places of the plot from ground level to the top of the spike (awns were not considered) at ripening before fully ripe stage	cm
Thousand kernel weight	TKW	Measured as the average of two samples of 500 kernels	g
Grain protein content	GPC	Determined by NIT or NIR spectrometers	%
Test weight	TW		kg hl^−1^

### 2.3 Weather conditions

**Figure 1:**
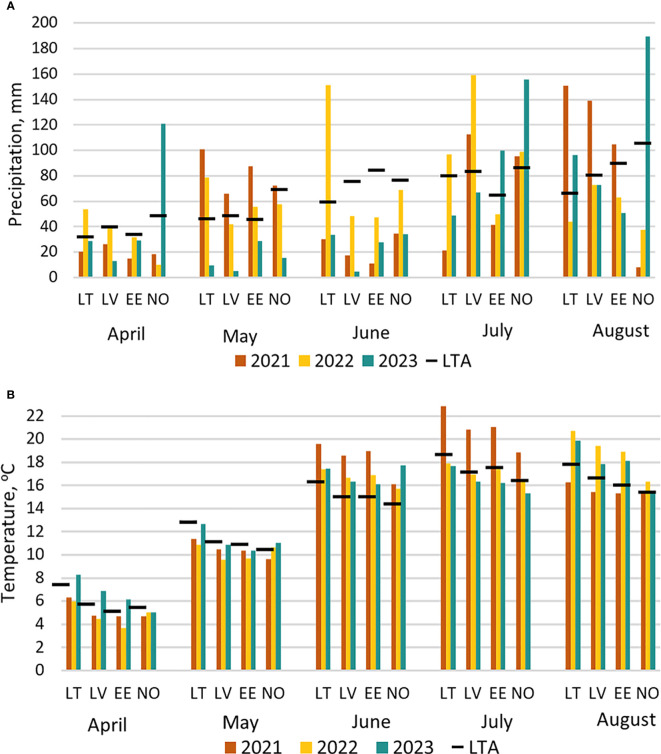
Meteorological conditions from April till August in three seasons (2021, 2022, 2023) in four study locations (LT, Lithuania; LV, Latvia; EE, Estonia; NO, Norway) compared to 30-year long-term average (LTA) data (1991–2020): **(A)** amount of monthly precipitation, **(B)** average air temperature.

Meteorological data across three growing seasons was collected from the nearest meteorological station to each of the four experimental sites (**Figure 1**). In general, lack of precipitation and high temperatures prevailed in 2021 and 2023, whereas 2022 had moderate temperatures along with an excess of precipitation. Due to heavy rains and partial flooding of the trial site in Lithuania in 2022, 52 trial plots were excluded from the analysis.

### 2.4 Phenotypic data analysis

For the examined phenotypic traits, adjusted means were calculated for each genotype in each tested trial (location and year combination) as well as global adjusted genotype means by using all available data from all tested trials according to design effects. To account for the possible inconsistency of the trial sites, genotypes were treated as fixed effects while trial columns, rows, and blocks were introduced as random effects into the model. Adjusted means were calculated with *lme4* (Bates et al., 2015) package for R (R Core Team, 2022). Broad-sense heritability (H^2^) was estimated using an extended Equation 2:


(2) $$\frac{var\left(genotype\right)}{\begin{array}{c} var(genotype)+\frac{var(genotype:location)}{m}+\frac{var(genotype:year)}{n}+\frac{var(Residual)}{mn} \end{array}}$$

Where *m* is the number of locations, and *n* is the number of years. The formula includes the interactions between genotypes and different environmental conditions across locations and years.

Best linear unbiased estimates (BLUEs) were calculated for each genotype for all tested traits for each trial site. Pearson’s correlation coefficients were estimated using R package “PerformanceAnalytics” (Peterson et al., 2020). The groups were compared using a Wilcoxon and Kruskal-Wallis tests. Data was visualized using R packages “ggplot2” (Wickham, 2016), “ggpubr” (Kassambara, 2023), and “PerformanceAnalytics.”

### 2.5 Plant genotyping, population structure, GWAS, and candidate gene identification

Genotyping of the plant material was performed as described earlier (Lin et al., 2024) under the frame of NOBAL wheat project. Raw marker data generated in the previous study was obtained; however, genotypic data curation and quality control were performed separately in this study. A subset of 18,467 high-quality genetic markers with less than 20% missing data and minor allele frequency (MAF) over 0.05 was selected for further analysis.

Population structure was determined by principal component analysis (PCA). For PCA, the missing genotypic data was imputed using the mean imputation method in *rrBLUP* package for R (Endelman, 2011). PCA was conducted using *“svd”* function from *base* package for R. Hierarchical clustering and the assignment of the genotypes to clusters was performed using *FactoMineR* package (Lê et al., 2008) and the dendrograms were drawn using *factoextra* package (Kassambara and Mundt, 2020) for R.

GWAS was performed using a Bayesian-information and Linkage-disequilibrium Iteratively Nested Keyway (BLINK) algorithm (Huang et al., 2019), designed to have high statistical power, and implemented into GAPIT (version 3) (Wang and Zhang, 2021) for R. A correction for population stratification was carried out by fitting the first five principal components (PCs) to the model to minimize occurrence of false positive associations. The optimal number of PCs was determined by analyzing PCA scree plot.

For GWAS, the *p*-value correction for multiple testing was performed using Bonferroni approach. Associations were considered significant if adjusted *p*-values were lower than 0.01 after correction for multiple testing. Statistical power analysis of BLINK method on genotypic data collected during this study was performed using GAPIT given high (H^2 = ^0.9) and average (H^2 = ^0.5) broad-sense heritability, with number of QTL set to three, and number of replications set to 10.

The percentage of phenotypic variance explained (PVE) by the markers was calculated by dividing their corresponding variance by the total variance (the sum of residual variance and the variance of the associated markers) (Wang and Zhang, 2021).

GWAS was performed on globally adjusted trait means and separately for each distinct trial to identify consistent or trial-specific markers. The marker was considered consistent if it was significant at least over two trials.

Identification of candidate genes putatively associated with the trait was carried out by screening ±150 kb genomic region from the significant marker. Gene names located in this region were extracted from the published spring wheat “Chinese Spring” RefSeq Annotation v1.1 (International Wheat Genome Sequencing Consortium (IWGSC), 2018) along with the distance from the marker using custom R scripts. Functional annotations for the identified gene models were inferred with DAVID tool (Sherman et al., 2022).

## 3 Results

### 3.1 Phenotypic variation

Overall, the NOBAL wheat spring wheat collection demonstrated high phenotypic variability across 12 trials (3 years and four locations) (**Table 2**). All main effects such as genotype, trial, and their interaction, were highly significant (*p*< 0.0001) for all studied traits. The trials had a major effect on the variation of tested traits, as reflected by high sums of squares due to contrasting environmental conditions, whereas the genotype effect was less pronounced. The estimated broad-sense heritability across 12 trials ranged from 0.79 to 0.95 for most studied traits, except for DH, where it was 0.5. The genotype effect on DH increased when the temperature accumulation was considered and growing degree-days (GDD) from sowing to heading were calculated. The trial effect was further reduced when PTUs that consider the effect of day length at each location were taken into account (**Table 2**).

**Table 2:** Basic phenotypic characteristics of the main agronomic traits, genotype x environment interaction (GEI), and broad-sense heritability across four locations and three years.

Trait	Min	Max	Mean ± SD	*F*-values			Explained (%) ◊				H^2^
				Gen	Env	GxE	Gen	Env	GxE	Resid.	
GY	315.42	615.83	510.42 ± 133.41	15.35^*^	1433.52^*^	1.32^*^	16.22	55.92	15.35	12.50	0.79
PH	63.44	101.15	78.60 ± 15.02	29.95^*^	5414.90^*^	1.58^*^	11.56	77.15	6.72	4.58	0.83
TKW	29.26	47.83	37.87 ± 4.28	78.55^*^	2793.38^*^	2.45^*^	35.64	46.79	12.22	5.35	0.90
DH	56.79	65.63	60.41 ± 6.42	117.69^*^	47484.39^*^	2.77^*^	6.15	91.63	1.59	0.63	0.50
GDD	417.42	546.87	472.14 ± 58.88	110.93^*^	16825.21^*^	2.71^*^	14.34	80.26	3.85	1.55	0.89
PTU	410.21	617.55	494.99 ± 83.71	110.69^*^	12535.50^*^	2.78^*^	17.97	75.11	4.97	1.95	0.95
MAT	100.73	110.64	105.12 ± 7.06	21.45^*^	10014.24^*^	2.52^*^	5.45	85.34	6.41	2.80	0.41
GPC	12.35	17.46	13.94 ± 2.34	43.05^*^	6452.89^*^	2.03^*^	13.65	75.53	7.07	3.75	0.80
TW	73.52	81.28	77.87 ± 3.33	27.75^*^	3514.02^*^	2.54^*^	16.66	63.70	13.73	5.91	0.81

SD, standard deviation; GxE, genotype × environment interaction; Gen, genotype; Env, environment (trial); Resid., residuals; H^2^, broad-sense heritability.

GY, grain yield; PH, plant height; TKW, thousand kernel weight; DH, days to heading; MAT, days to maturity; GDD, growing degree days; PTU, photo thermal units; GPC, grain protein content; TW, test weight.

^*^ All p-values below 0.0001.

◊ explained variation according to the sum of squares.

The highest average GY was obtained in 2022, while lower average GYs were recorded in 2021 and 2023 at all locations except Lithuania. The average GY of 299 genotypes over three years and four locations ranged from 341.2 to 718.2 g m^−2^ (**Supplementary Table S3**). The highest average GY was recorded in Estonia in 2022, while the lowest GY was observed in Lithuania in 2023. The most yielding genotype was DS-674–9-DH (NW293), with an average yield of 615.8 g m^−2^, while Sumai #3 (NW045) had the lowest yield of 315.4 g m^−2^ across all trials.

Over 12 trials, the highest average GPC was 17.4% in Estonia in 2023, while the lowest average of only 9.7% was reached in Norway in 2021 (**Supplementary Table S3**). The genotype Sport (NW037) had the highest average GPC of 17.5% across all 12 trials. The outliers exhibited extremely high GPC (**Supplementary Figure S1**).

The average values of thousand kernel weight (TKW) ranged from 32.3 to 43.6 g across studied trials (**Supplementary Table S3**). The highest average values were obtained in 2022 across all locations. Additionally, high average values of TKW were observed in Lithuania, Latvia, and Estonia in 2023 under conditions where plants experienced drought during tillering. Three-year average TKW was highest in Norway in 2022 and lowest in Lithuania in 2021. The genotype DS-732–5-DH (NW276) had the highest TKW across four locations and 3 years, while the lowest TKW was observed for the genotype Sport (NW037) (**Supplementary Figure S1**).

Across 12 trials, the average PH varied from 46.6 to 95.5 cm. The shortest PH was observed in Estonia in 2023, while the tallest was recorded in Latvia in 2022 (**Supplementary Table S3**). Irsijevskaja (NW148) was the tallest genotype, averaging 101.2 cm across 12 trials, while Caral (NW206) was the shortest, averaging only 63.4 cm across all trials (**Supplementary Figure S1**).

Across 10 trials (the trait was not scored in Norway in 2021 and 2022), the average values of TW varied from 72.6 to 82.1 hl^−1^ (**Supplementary Table S3**). Vanek (NW236) had the highest TW of 81.3 kg hl^−1^ on average across 12 trials, while Serenada (NW228) had the smallest TW of 73.5 kg hl^−1^.

The shortest duration from sowing till heading, expressed as GDD, was observed in Latvia in 2023, while the longest GDD was recorded in Lithuania in 2021. Across 12 trials, the average GDD varied from 376.8 to 584.9. DS-530–10-DH (NW258) was the latest-heading genotype, with 546.9 GDDs, while Kanyuk (NW119) was the shortest-heading, with only 417.4 GDD on average. The distinct shapes of the violin plots for the number of days from sowing till heading, as well as for GDD, indicate differences in growing trials that had major impact on this trait (**Supplementary Figure S1**).

Across 12 trials, the average number of MAT varied from 98.97 to 117.3 days. The shortest MAT was observed in Estonia in 2021, while the longest was recorded in Latvia in 2022. Cultivar Manu (NW088) had the shortest length of the growing period, with only an average of 101.92 days, and breeding line DS-638–5-DH (NW262) had the longest length of the growing period, with 111.29 days on average. 

**Figure 2:**
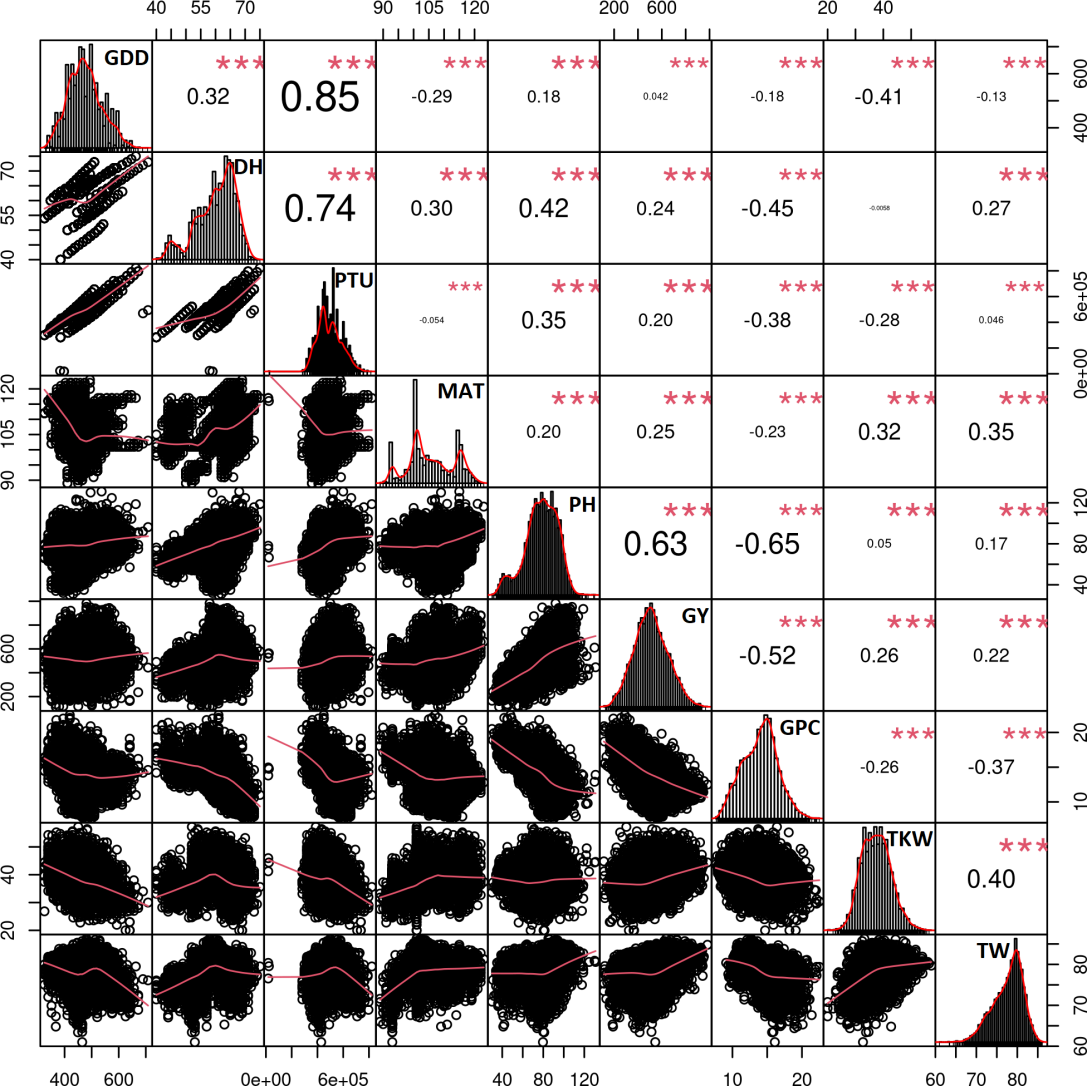
Correlation matrix between the traits using the data from all trials and includes the data from 7,148 plots. All correlations were highly significant with the *p*-values< 0.001 as indicated by ***. Trait histograms are provided diagonally, while the scatterplots can be observed on the lower left part of the plot. GDD, growing degree days to heading; DH, heading date; PTU, photothermal units to heading; MAT, maturity date; PH, plant height; GY, grain yield; GPC, grain protein content; TKW, thousand kernel weight; TW, test weight.

Highly significant correlations were observed between all examined traits when pooling data from a total of 7148 trial plots (**Figure 2**). PH strongly and positively correlated with the GY (*r* = 0.63). GCP displayed negative correlations with PH (*r* = −0.65) and GY (*r* = −0.51). Furthermore, a moderately strong correlation was observed between TW and TKW (*r* = 0.4) and slight but significant correlation between TKW and GY (*r* = 0.25). Trial-specific correlations between the traits can be found in **Supplementary Figures S2**–**S13**.

### 3.2 Population structure

**Figure 3:**
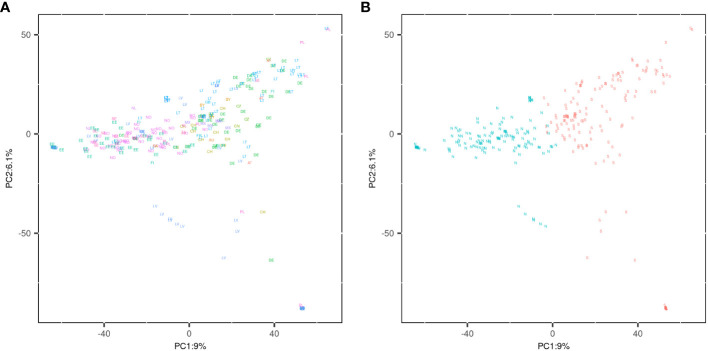
**(A)** PCA plot demonstrating the distribution of 299 spring wheat genotypes using 18,467 polymorphic SNPs. The countries of genotype origin are encoded using an ISO 3166–1 alpha-2 two-letter country codes and distinct colors. **(B)** PCA plot according to the genotype clustering information. “N” letter represents genotypes found in the Northern cluster, while “S” represents genotypes in Southern cluster.

The first two PCs explained 9.3% and 5.9% of the total genetic variance within NOBAL wheat spring wheat panel, respectively (**Figure 3A**). Based on 18,467 genetic markers, spring wheat accessions can be grouped according to the region of origin. PC1 coordinate separated most of the accessions developed in the Nordic countries and Estonia from the accessions developed in other Baltic, Central and Western Europe countries. Interestingly, some exotic, Mexican accessions from The International Maize and Wheat Improvement Center (CIMMYT) did not form a separate cluster and were closely linked to the genotypes developed in the Baltic region. Nevertheless, there was a clear separation of some Latvian accessions on the PC2.

Genotypes were assigned into two clusters (**Figure 3B**) by hierarchical clustering on principle components implemented in *FactoMineR* package. The Northern cluster (136 genotypes) was dominated by the Norwegian (38), Estonian (34), and some Latvian (24) genotypes. The Southern cluster (163 genotypes) was comprised mainly of the Lithuanian (48), German (38) and Latvian (27) genotypes. The full list of genotypes in Northern and Southern clusters is provided in **Supplementary Table S1** and **Supplementary Table S4**. Interestingly, the genotypes of Latvian origin almost equally distributed between these two clusters.

To identify the genetic loci associated with differentiation of Northern and Southern genotype clusters, we encoded the clusters as binary ‘dummy’ phenotypes and conducted GWAS with no correction for population structure. Manhattan and Q-Q plots are provided in **Supplementary Figure S14**. A GWAS yielded seven significant markers likely associated with the divergence of these clusters in NOBAL wheat collection. The most notable and significant marker BobWhite_c33344_143 was located on chromosome 3B, within the *TraesCS3B01G490600* gene, which encodes glutathione S-transferase 3-like. The MAF for this most significant marker was 0.43 in the whole NOBAL wheat association panel. However, in the Northern cluster the T allele was dominating with the frequency of 0.83, and quite opposite, in the Southern cluster the T allele was rarely detected with the frequency of only 0.1. Similarly, the minor alleles for AX-158543919 and AX-158559797 (chr 7A) markers were also common in Northern genotype cluster compared to the Southern genotypes with the frequency of 0.78 and 0.82, respectively. However, the minor allele for AX-95175904 (chr 3D) was rarely observed in the Northern genotypes. The full list of significant genetic markers associated with the differentiation of genotype clusters can be found in **Supplementary Table S5**.

**Figure 4:**
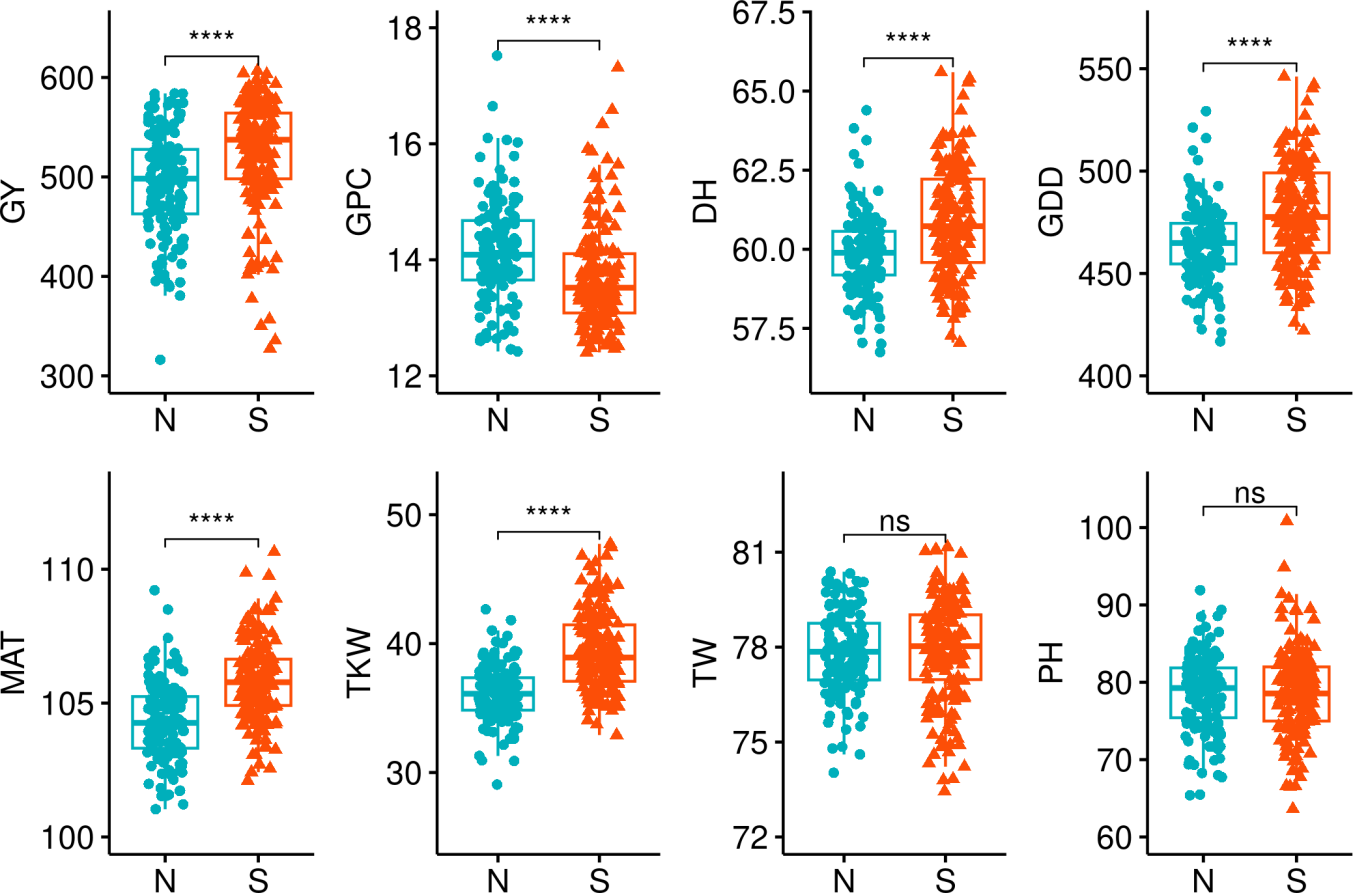
Phenotypic traits (BLUEs) comparison between the Northern and Southern spring wheat genotype clusters. The clusters were compared using Wilcoxon test. **** denotes *p*-values< 0.001, ns, not significant; GY, grain yield; GPC, grain protein content; DH, days to heading; GDD, growing degree days; MAT, maturity date; TKW, thousand kernel weight; TW, test weight; PH, plant height.

**Figure 5:**
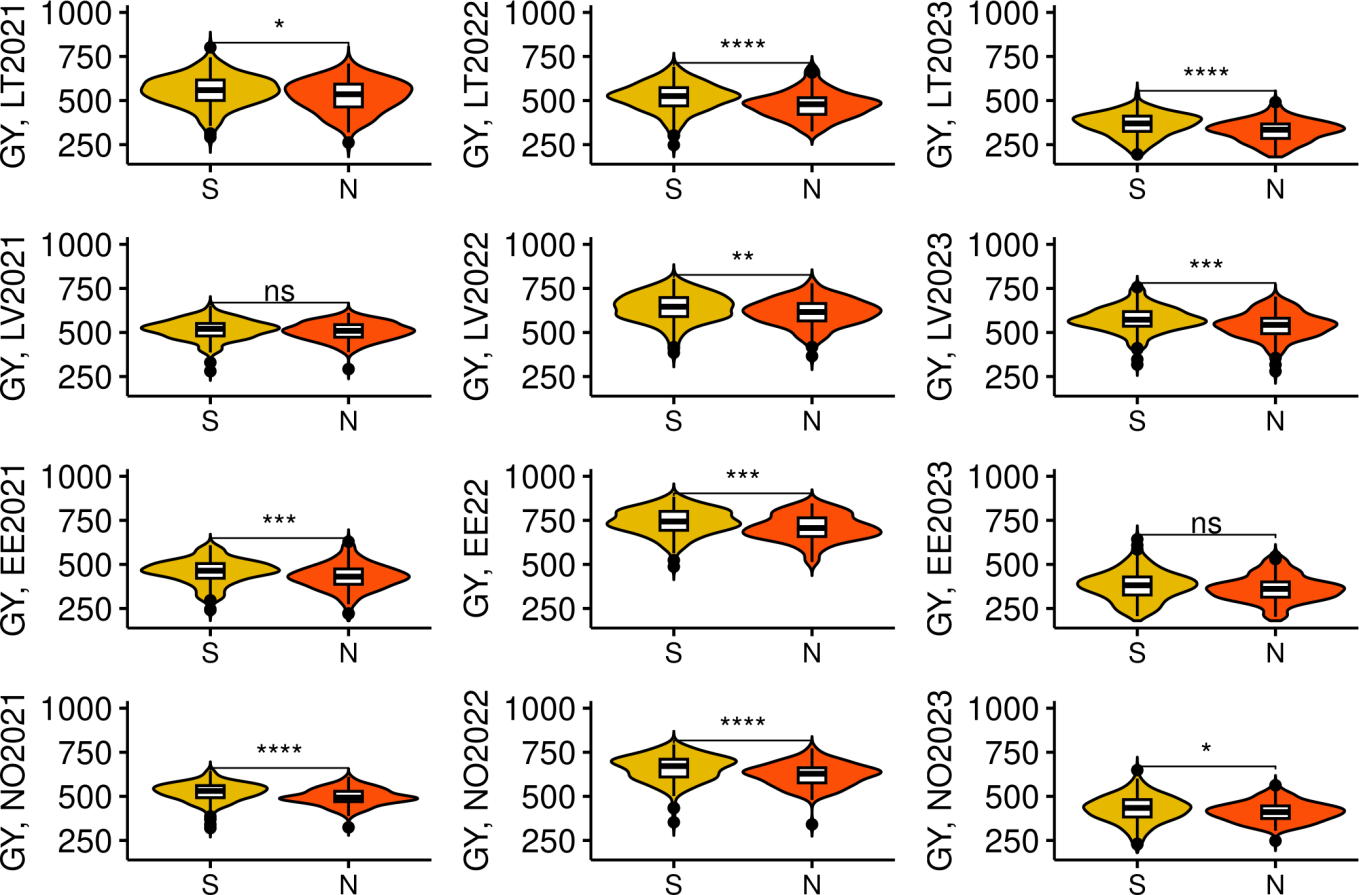
Grain yield (GY) performance of spring wheat lines in Northern and Southern genotype clusters in multiple trials. “S” and “N” denote Southern and Northern genotype clusters respectively. The trial sites were abbreviated as following: LT, Lithuania; LV, Latvia; EE, Estonia; and NO, Norway. The year of the trial is provided next to the site name. *, **, ***, and **** denote p-values of < 0.05, 0.01, 0.001, and 0.0001, respectively, while ns - not significant.

Some significant differences (*p*< 0.01) in the examined traits were observed between the genotypes in the Northern and Southern clusters. (**Figure 4**). We compared trait BLUEs, and evidently, genotypes in the Northern cluster were on average lower yielding, exhibited higher GPC, headed and matured earlier, with a TKW significantly lower compared to genotypes in Southern cluster. However, there was no significant difference between these two clusters in grain TW and PH. It is expected for local varieties to be adapted to local climate to maximize yield potential. Nevertheless, the yield performance of genotypes in the Northern cluster was significantly lower on average (*p*< 0.05) during all tested years and locations, including Norway (**Figure 5**).

Tight clustering was evident on the kinship matrix heatplot, indicating a close genetic relatedness between some of NOBAL wheat accessions (**Supplementary Figure S15**). The most notable cluster of these accessions comprised mostly Latvian accessions, except a single accession from Estonia. In general, such clusters of closely related accessions were dominated by the genotypes originating from distinct countries/breeding programs. Despite some clear clustering, the kinship between other accessions seemed to be quite low in general.

### 3.3 Genome-wide association study

Previously generated genotypic data (Lin et al., 2024) of the 299 accessions genotyped with 25K SNP array was used to perform GWAS. After quality check and filtering, a total of 18,467 genetic markers were extracted for GWAS. A summary of genetic marker mapping information is provided in **Supplementary Table S6**. GWAS was conducted for each trait in twelve trials separately, except for TW, which was not scored in Norway in year 2021 and 2022. Distinct trials represented year-location combinations. The corresponding Manhattan and quantile–quantile (Q-Q) plots are provided in the supplementary information (**Supplementary Figures S16**–**S31**). GWAS statistical power analysis is presented in **Supplementary Figure S32**. QTL significant in two or more trials were considered as consistent. In this study, only consistent QTL are discussed, omitting trial-specific associations. Genetic markers which consistently explained less than 5% of phenotypic variation are excluded from further analysis. The functional annotations for the genes identified in the QTL intervals are provided in the **Supplementary File S1**.

### 3.4 Marker-trait associations for plant height

**Figure 6:**
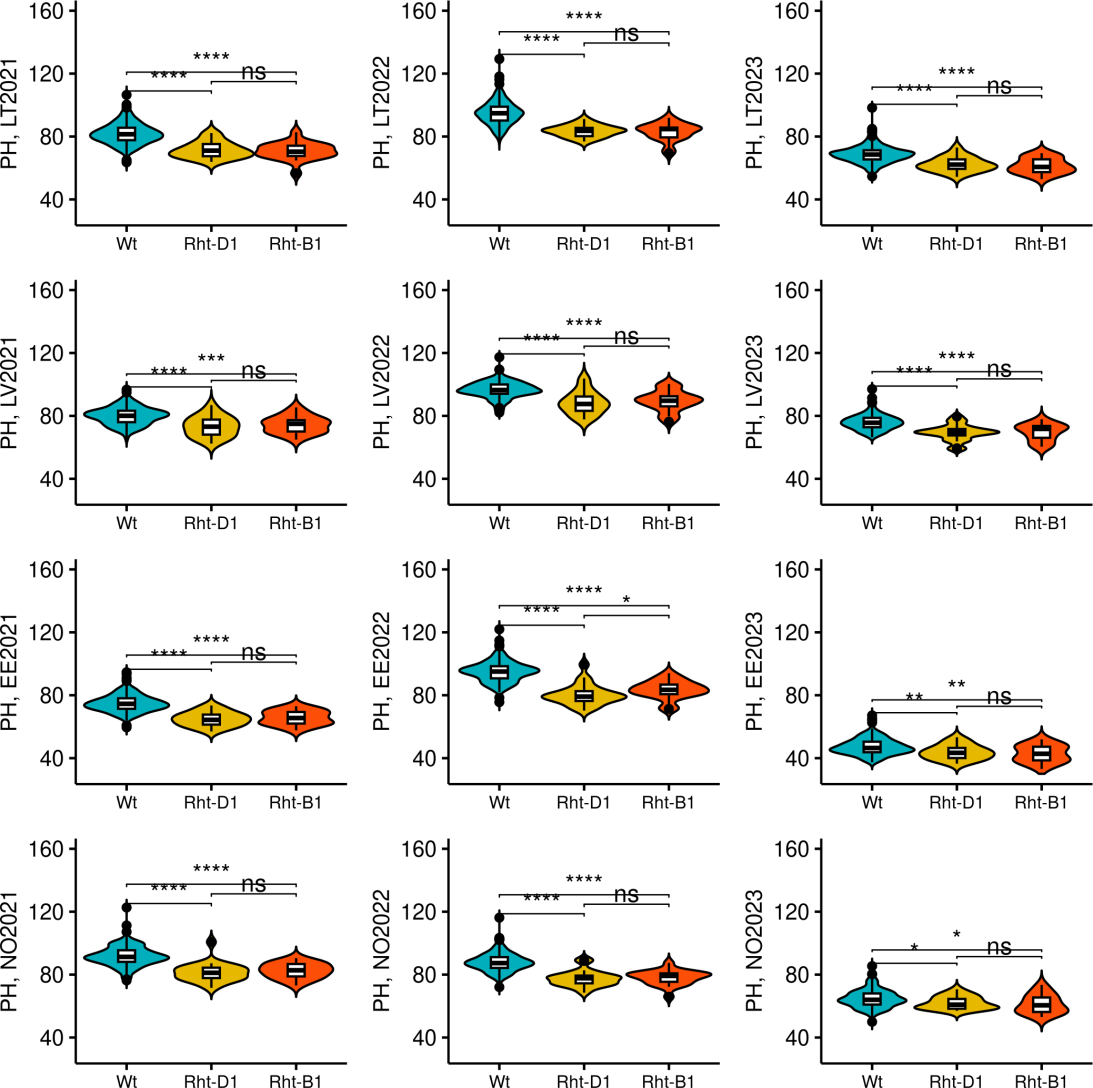
The effect of *Rht-B1* and *Rht-D1* alleles on plant height (PH). *Wt* genotypes denotes genotypes harboring no semi-dwarfism-related *Rht* alleles. The genotypes were compared using Kruskal–Wallis test. The trial sites were abbreviated as following: LT, Lithuania; LV, Latvia; EE, Estonia; and NO, Norway. The year of the trial is provided next to the site name. *, **, ***, and **** denote p-values of < 0.05, 0.01, 0.001, and 0.0001, respectively, while ns - not significant.

For the PH, four identified QTL were consistent and highly significant. The most consistent markers for PH were TG0010a (chr 4B) and TG0011a (chr 4D), and were detected in five and eight trials out of 12 tested, respectively (**Table 3**). In addition, the highest proportion for phenotypic variation for PH was attributed to these two markers and explained up to 17.95% and 29.16% of observed phenotypic variation, respectively. The minor TG0010a and TG0011a alleles were associated with a significantly lower PH (*p*< 0.05), compared to major (wild type) alleles in all tested trials. However, no significant differences in PH were detected between the genotypes which harbored TG0010a or TG0011a minor alleles, except for Estonia in 2022, where the accessions harboring TG0010a minor allele were taller on average (*p*< 0.05) than the accessions with TG0011a minor allele (**Figure 6**). Candidate genes in the QTL intervals associated with PH are provided in **Supplementary Table S7**. The TG0010a and TG0011a markers were located within the gene models which encode well-known proteins associated with PH in wheat. The marker TG0011a resided on wheat chromosome 4D in *TraesCS4D02G040400* gene, while TG0010a was located on chromosome 4B within the *TraesCS4B02G043100* gene. The sequence analysis of these genes revealed that *TraesCS4D02G040400* encodes DELLA protein Rht-D1, while *TraesCS4B02G043100* encodes DELLA protein Rht-B1. Reduced height (*Rht*) genes are known to cause limited response to the phytohormone gibberellin and semi-dwarfism. Allele substitution at TG0010a site from C to T leads to the introduction of premature stop codon (Q60Stop) in DELLA protein Rht-B1. Therefore, because of allele substitution at the site TG0010a, semi-dwarf genotypes encode severely truncated DELLA protein Rht-B1 (**Supplementary Figure S33**). For DELLA protein Rht-D1, nucleotide substitution at the site TG0011a from G to T leads to Glu -> Asp (E64D) amino acid substitution in the encoded protein (**Supplementary Figure S34**).

**Table 3:** Significant genetic markers identified by GWAS associated with plant height.

Marker	Chr	Position (Mbp)	MAF	Phenotypic variance explained (%)	Trial
wsnp_Ex_c7965_13520238	1A	12.37	0.07	4.46–5.00	EE21, LV23
wsnp_JD_c30422_23944042	3B	100.97	0.15	2.59–5.88	EE22, LT23
TG0010a	4B	30.86	0.08	6.31–17.95	EE21, EE22, LT21, LV23, NO22
TG0011a	4D	18.78	0.07	16.72–29.16	EE21, EE22, LT21, LT22, LV22, LV23, NO21, NO22

Chr, chromosome; MAF, minor allele frequency.

Physical positions of the markers are given for Wheat Chinese Spring IWGSC RefSeq v1.0 genome assembly.

The trial abbreviations are as the following - letters denote locations, while the digits denote years: EE, Estonia; LT, Lithuania; LV, Latvia; NO, Norway; 21, 22, and 23 = year 2021, 2022, and 2023, respectively.

**Figure 7:**
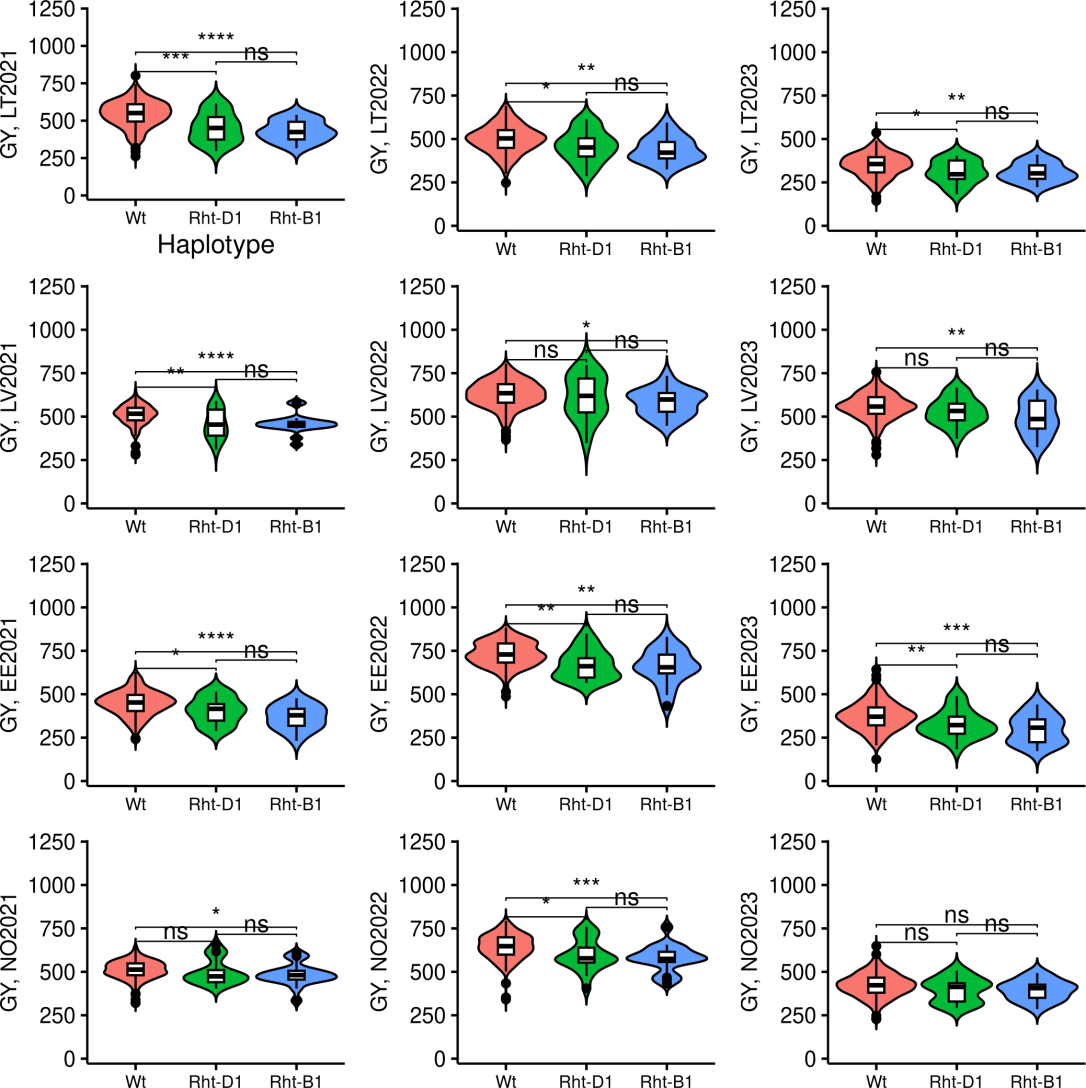
The effect of *Rht-B1* and *Rht-D1* alleles on grain yield (GY). *Wt* genotypes denotes genotypes harboring no semi-dwarfism-related *Rht* alleles. The genotypes were compared using Kruskal–Wallis test. The trial sites were abbreviated as following: LT, Lithuania; LV, Latvia; EE, Estonia; and NO, Norway. The year of the trial is provided next to the site name. *, **, ***, and **** denote p-values of < 0.05, 0.01, 0.001, and 0.0001, respectively, while ns - not significant.

The frequencies of minor alleles associated with semi-dwarfism for TG0011a and TG0010a in the panel were 0.07 and 0.075, respectively. Thus, minor alleles for these markers were not common in the studied panel, where only 19 genotypes had TG0010a (T) allele, while 21 genotype possessed TG0011a (T) allele. No genotypes harboring both TG0010a and TG0011a minor alleles were identified. The minor T allele of TG0010a was more common in the Norwegian origin (nine accessions) than in Estonian (one accession) or Latvian (two accessions) genotypes. TG0011a minor allele was also more common in Norwegian genotypes (12 accessions), compared to those from Estonia (one accession), and Lithuania (three accessions). The genotypes in NOBAL wheat association panel which harbored *Rht* semi-dwarf alleles were generally lower yielding in most of the tested trials (**Figure 7**). However, in 2023, no yield difference between wild-type (*Wt*) and semi-dwarf *Rht* alleles-bearing genotypes was observed in Norway. Moreover, semi-dwarf *Rht-B1* allele bearing genotypes were significantly lower yielding compared to *Wt* in other trials, while *Rht-D1* genotypes did not significantly differ from *Wt* in LV2022, LV2023, and NO2021 trials. Furthermore, there was no significant difference in GY between the genotypes with *Rht-B1* and *Rht-D1* dwarfing alleles in all tested trials.

The wsnp_JD_c30422_23944042 locus which was significantly associated with PH in Estonian trial in 2022 and Lithuanian in 2023 defines a genetic region on chromosome 3B. Identified QTL explained up to 5.88% of phenotypic variation for PH and resides within *TraesCS3B01G125400* gene model with a high sequence homology to *NDL1*-like gene which was earlier associated with plant hormone auxin signaling.

### 3.5 Marker-trait associations with grain yield and grain quality-related traits

Fourteen trial-specific marker-trait associations (MTAs) with GY were detected in all tested trials, except for Latvia in 2022; however, we did not detect any markers consistently associated with the GY in the NOBAL wheat association panel.

For GPC, we identified three consistent QTL explaining more than 5% of the variation. Ku_c12842_664 (chromosome 1D) and Excalibur_c5700_244 (chromosome 7B) were significantly associated with GPC in three trials, while Kukri_c57014_168 (chromosome 2B) was detected in two different trials. The markers explained up to 13.76% of observed GPC variation within the panel (**Table 4**). Ku_c12842_664 and Kukri_c57014_168 markers were directly located within the genes *TraesCS1D02G031300* and *TraesCS2B02G538900*, respectively. However, Excalibur_c5700_244 was in intergenic space where the neighboring genes were 7 and 9 kb away from the marker.

**Table 4:** Significant genetic markers identified by GWAS associated with grain quality-related traits.

Trait	Marker	Chr	Position (Mbp)	MAF	Phenotypic variance explained (%)	Trial
GPC	Ku_c12842_664	1D	12.30	0.05	7.31–13.76	LT21, LT23, NO22
	Kukri_c57014_168	2B	734.13	0.24	4.39–10.21	LV21, LV22
	AX-158542602	5A	689.89	0.12	6.19–8.15	EE21, NO21
	Excalibur_c5700_244	7B	608.91	0.05	6.79–10.24	EE22, LT21, LV21
TKW	BS00074911_51	1B	626.23	0.09	4.00–7.66	LV22, NO22, NO23
	AX-94858447	4A	715.75	0.06	4.69–7.37	EE22, LT22
	AX-86185201	6B	103.29	0.07	4.99–13.38	EE22, LT21, LV22
	AX-158556233	7A	671.78	0.20	2.24–20.57	LV21, LV23, NO21, NO22, NO23
TW	Excalibur_rep_c101263_892	2B	18.94	0.13	14.93–29.55	LT23, LV22
	IAAV6424	2B	691.78	0.35	10.83–18.90	LT21, LT22, LV21
	TGWA25K-TG0157	UN	NA	0.13	5.29–15.36	EE22, LT21

Chr, chromosome; MAF, minor allele frequency; GCP, grain protein content; TKW, thousand kernel weight; TW, test weight.

Physical positions of the markers are given for Wheat Chinese Spring IWGSC RefSeq v1.0 genome assembly. The trial abbreviations are as the following - letters denote locations, while the digits denote years: EE, Estonia; LT, Lithuania; LV, Latvia; NO, Norway; 21, 22, and 23 – year 2021, 2022, and 2023, respectively.

For TKW, we have identified four consistent QTL, which explained up to 20.54% of variation for TKW. The MTA for the AX-158556233 marker (chromosome 7A) was the most consistent and explained the largest proportion of phenotypic variation. This association was significant in five trials and explained 2.24%–20.57% of variation for TKW (**Table 4**). The marker was located next to the *TraesCS7A02G479000* encoding cyclin-D5–3-like protein. The two other markers associated with TKW, AX-86185201 (chromosome 6B) and BS00074911_51 (chromosome 1B), were detected in three trials, while AX-94858447 MTA was detected in two trials. AX-86185201 was located on chromosome’s 6B gene-scarce intergenic space, while BS00074911_51 (chromosome 1B) and AX-94858447 (chromosome 4A) were in *TraesCS1B02G393400* in *TraesCS4A02G449800*, respectively. Moreover, genotypes harboring minor alleles for AX-158556233 and AX-86185201 had significantly (*p*< 0.01) higher TKW on average than genotypes with major alleles in all tested trials (**Supplementary Figures S35**, **S36**).

Three consistent MTAs with grain TW were identified. Excalibur_rep_c101263_892 and TGWA25K-TG0157 were significant in two trials, while IAAV6424 was significant in three trials. These three markers were located within genes *TraesCS2B02G041600*, *TraesCSU02G000100*, and *TraesCS2B02G494800*, respectively. The minor allele of IAAV6424 was moderately common in the NOBAL wheat panel with the MAF of 0.35, while MAF for the other two markers was 0.13. The markers explained from 5.29% to 29.55% of observed phenotypic variation for TW, depending on marker and trial (**Table 4**).

### 3.6 Marker-trait association for heading date-related traits and days to maturity

To detect QTL associated with the heading date, we conducted GWAS on two heading date-related traits: DH and temperature adjusted GDDs to heading.

For DH, we detected 8 consistent QTL, however, only three of these explained more than 5% of the phenotypic variation for DH. These three QTL explained up to 7.27%–20.71% of the variation for DH (**Table 5**). AX-109946561 marker with MAF of 0.37 was the most consistent and was detected as significant in five trials and explained up to 20.71% of the phenotypic variation in NOBAL wheat panel. The marker was in an intergenic space in a gene-rich region with nine gene models detected within the QTL interval. The other two significant markers, AX-95099408 and BS00067975_51, were associated with two distinct trials and explained up to 7.27% and 20.5% of DH variance, respectively. AX-95099408 marker resided within *TraesCS4B02G000500* gene for ACT domain-containing ACR10-like protein, while BS00067975_51 was mapped into the intergenic space. *TraesCS2B02G140300* was detected in the BS00067975_51 QTL region with high sequence similarity to a gene encoding zinc finger protein CONSTANS-LIKE 13-like.

**Table 5:** Significant genetic markers identified by GWAS associated with heading date-related traits.

Trait	Marker	Chr	Position (Mbp)	MAF	Phenotypic variance explained (%)	Trial
GDD	AX-109946561	1B	57.63	0.37	2.17–18.83	EE22, EE23, LT23, LV23, NO21, NO22
	BS00067975_51	2B	107.08	0.06	11.64–27.63	EE21, EE22
	AX-158523146	3A	741.62	0.48	1.04–10.28	EE21, LT22, LT23
	AX-95099408	4B	0.54	0.13	2.21–7.20	LT23, NO21
	BobWhite_c26680_62	5B	13.18	0.06	5.98–22.05	EE23, LV23
DH	AX-109946561	1B	57.63	0.37	5.01–20.71	EE22, EE23, LV23, NO21, NO22
	BS00067975_51	2B	107.08	0.06	12.31–20.50	EE21, EE22
	AX-95099408	4B	0.54	0.13	1.94–7.27	EE22, NO21
PTU	AX-109946561	1B	57.63	0.37	5.04–9.63	EE22, LT22, NO21, NO22

Chr, chromosome; MAF, minor allele frequency; GDD, growing degree days to heading; DH, days to heading; PTU, photothermal units to heading.

Physical positions of the markers are given for Wheat Chinese Spring IWGSC RefSeq v1.0 genome assembly. The trial abbreviations are as the following - letters denote locations, while the digits denote years: EE, Estonia; LT, Lithuania; LV, Latvia; NO, Norway; 21, 22, and 23 = year 2021, 2022, and 2023, respectively.

Several markers were significantly associated with GDD. As for DH, the same set of consistent significant markers was detected. However, there were two additional consistent associations with the markers AX-158523146 (chromosome 3A) and BobWhite_c26680_62 (chromosome 5B), which explained up to 10.28% and 27.63% of GDD variation, respectively. The marker AX-158523146 was located next to *TraesCS3A02G527200*, while BobWhite_c26680_62 close to *TraesCS5B01G013200* and *TraesCS5B01G013300* genes.

No consistent markers were identified for the MAT, which could explain more than 5% phenotypic variance for this trait.

## 4 Discussion

Climate warming is occurring at a rapid pace at Northern latitudes; therefore, we urgently need wheat cultivars better adapted to shifting conditions to mitigate the impact of climate change on wheat production. A diverse collection of spring wheat genotypes was tested at 12 trials (three seasons at four locations) and demonstrated overall plasticity and potential for beneficial alleles. Phenotypic data collected during 2021 and 2022 field seasons described by Ingver et al. (2023) was supplemented with additional information from 2023 growing season. A lack of precipitation combined with elevated temperatures was recorded in two out of three seasons at all four locations. These weather extremes occurred during critical stages of plant development from tillering to flowering. Such shifts in precipitation are also predicted by climate models (Pokhrel et al., 2021) and will inevitably have a pronounced effect on GY in spring crops as was noted in this study. Therefore, results presented in this study are a valuable resource for breeders.

However, even at these unfavorable conditions a high variability in GY was detected among genotypes with some expressing higher adaptability to low precipitation and elevated temperatures. GY is a complex trait representing an interaction of multitude of wheat developmental traits with environmental conditions. In our study, PH had the highest correlation with GY where taller genotypes were also better yielding. Genome-wide association analysis revealed four consistent QTL for PH with two QTL on chromosomes 4B and 4D having the highest effect across most of the trials. These two QTL represent *Rht* (*Reduced-height*) genes harboring mutant semi-dwarf alleles *Rht-B1b (Rht-1)* and *Rht-D1b (Rht-2)*. *Rht* genes are so-called Green Revolution genes as they confer thicker and shorter stems which enables the development of non-lodging semi-dwarf wheats with better harvest index. The semi-dwarf mutant alleles were introduced into wheat breeding programs from Japanese wheat cv. Norin 10 in the early 1950’s and are well utilized in commercial wheat breeding programs world-wide (Jatayev et al., 2020). Surprisingly, in our NOBAL wheat spring wheat panel comprised of 299 genotypes, only 19 and 21 genotypes carried semi-dwarf *Rht-B1b* and *Rht-D1b* alleles, respectively, and no genotypes harboring both alleles were detected. According to Knopf et al. (2008), mutant alleles *Rht-B1b* and *Rht-D1b* were identified in 6% and 38% of German winter wheat cultivars, respectively. Similarly, *Rht-B1b* and *Rht-D1b* were detected in 7% and 58% of recently registered winter European cultivars, respectively (Zanke et al., 2014). In our study, the occurrence of *Rht-B1b* and *Rht-D1b* alleles was 6% and 7%, respectively. The majority of *Rht-B1b* and *Rht-D1b* alleles were found in genotypes originating from the Nordic countries. These results might be explained by the adverse effects of these mutant alleles under drought and by the higher drought susceptibility of spring wheat compared to winter wheat. Consequently, diminishing occurrence of these mutant alleles appears to be related to breeding for improved adaptability to drier conditions. Moreover, spring wheat genotypes carrying wild type *Rht* alleles were better yielding than genotypes with mutant alleles in most of the tested trials, especially under hot and dry conditions during tillering. The questionable benefits of *Rht* semi-dwarf alleles in hot and dry environments were recently discussed by (Jatayev et al., 2020). They concluded that dwarfing alleles are beneficial under well-irrigated wheat production systems with high nitrogen input; however, they are not well-suited under prolonged drought conditions. In dry conditions semi-dwarf plants suffer from early arrested growth, resulting in smaller plant biomass, fewer tillers and spikes, and limited grain production (Wang et al., 2015), while taller genotypes produce tillers earlier, accumulate carbohydrate stores and remobilize those to fill grains (Zhang et al., 2006). Nordic and Baltic countries are not regarded as arid environments, however, clear shift to drier late springs and early summers, as was recorded in this study, might reflect the low distribution of *Rht* semi-dwarf alleles in advanced breeding material. To date, a total of 25 *Rht* genes were documented in wheat with various effects on PH and are generally classified into two classes in response to gibberellic acid (GA) application, for example, GA-sensitive and GA-insensitive, with *Rht-B1* and *Rht-D1* belonging to GA-sensitive class. Other phytohormones, such as auxin, cytokinin and abscisic acid, also play crucial roles in controlling PH. In our study, QTL (wsnp_JD_c30422_23944042) on chromosome 3B tagged a NDL1-like gene associated with plant hormone auxin signaling (Chen and Gallavotti, 2021) while three additional QTL were identified to be associated with PH and might be valuable alternatives to semi-dwarf *Rht-B1b* and *Rht-D1b* alleles.

Historically, wheat GY improvements were mainly achieved through increased number of grains per area (Voss-Fels et al., 2019), whereas grain size measured as TKW was associated with little yield improvement despite its importance as grain quality determinant. Substantial variation for TKW in our spring wheat panel even at unfavorably dry growing conditions indicates a potential for genetic improvement of this trait. Four QTL for TKW were identified on various chromosomes with the most consistent ones on chromosomes 1B, 2A, 6B, and 7A.

GPC is a primary target trait for improvement in every wheat breeding program; however, tight negative correlation with GY hinders the progress (Geyer et al., 2022). Despite observing substantial variation for GPC among genotypes and environments the negative correlation with GY remained evident in our study. GWAS revealed four QTL for GPC on chromosomes 1D, 2B, 5A, and 7B. Noteworthy, chromosome 2B region was previously associated with GPC in a GWAS on 372 diverse European wheat varieties (Muqaddasi et al., 2020). The 2B QTL from our study co-located with GPC and yield QTL from the study of White et al. (2022).

Transition from vegetative to reproductive phase is one of the most critical stages in plant development. The timing and duration of the transition must be well synchronized with environmental conditions to ensure optimal exploitation of water, solar, and nutrient resources maximizing production of GY (Nazim Ud Dowla et al., 2018; Senapati et al., 2019; Shi et al., 2019). In the Baltic region, a vegetative period is longer and enables accumulation of larger amount of biomass, whereas this period is shorter at higher latitudes, such as Norway. The most common estimator of wheat earliness is the number of days from emergence to heading or anthesis. However, this simplification might be misleading when experiments are carried out across diverse environments and different years. These environmental irregularities should be accounted for when calibrating earliness data versus temperature or even day length, as was the case in our study, where experimental sites represented latitudinal cline. In general, both calibrations improved data reproducibility across environments with slightly better effect with temperature adjustment than day length adjustment. There are several molecular pathways regulating wheat transition from vegetative to regenerative phase including vernalization, photoperiod sensitivity and light perception pathways as well as *earliness-per-se* (*eps*) genes (Nazim Ud Dowla et al., 2018). Three major pathways were identified to interact during plant development and confer major shifts in developmental stage transition while *eps* genes were shown to fine-tune wheat earliness when vernalization and photoperiod requirements have been met (Snape et al., 2001). In our study, we have identified three QTL for wheat earliness when data was not corrected for temperature and day length. Two additional QTL emerged when temperature and photoperiod were considered. Interestingly one QTL contained a gene *TraesCS2B02G140300* with a high-sequence similarity to zinc finger protein CONSTANS-LIKE 13-like gene which was previously shown to control shoot branching and flowering in leafy *Brassica juncea* (Muntha et al., 2019). Early maturing spring wheat cultivars are usually desired at Northern latitudes due to the shorter growing season, and to avoid less favorable wet conditions at the end of the season causing deterioration of the wheat grain quality and delayed sowing of the aftercrop.

The genetic markers associated with agricultural traits and stability across multiple environments are of special interest. We propose several consistent markers identified in our study as candidates for implementation in marker-assisted selection of spring wheat. While the detected markers in *Rht* genes for PH were highly consistent, our results suggest that some additional consistent markers could be employed for the selection for heading date and thousand kernel weight.

Wheat production in Europe is predicted to shift to Northern latitudes due to climate change. This transition will be facilitated by longer vegetative seasons in Scandinavia and Baltic countries due to elevated temperatures, especially in autumn; however, shifts in precipitation regimes with drier spring-summer seasons will pose a risk for spring crops. Winter wheat will be the favored crop in the region due to its higher yield potential and better utilization of precipitation accumulated during the winter. However, spring wheat will be cultivated as well to fit into crop rotations and as a replacement crop for winter wheat during the seasons of impaired autumn sowing or poor overwintering. Thus, it is particularly important to develop spring wheat cultivars adapted to the predicted changes in growing conditions of the Northern European region. A PCA of the current spring wheat breeding material in Norway and the Baltic states revealed two clusters which we dubbed Northern and Southern, depending on the origin of most genotypes in the cluster. By examining allele frequencies in Northern and Southern sets, we discovered several marker alleles significantly associated with the sets. The finding might indicate that the genomic loci were under selection pressure due to adaptation to environmental conditions found in Nordic region or might indicate bias toward the selection of parents in crossings in national breeding programs. As expected, Northern genotypes were earlier, however lower yielding, had lower TKW and higher GPC. Moreover, during three consecutive years of study, the average GY performance of genotypes developed at southern latitudes was consistently higher compared to Northern genotypes even in Nordic growth conditions. Interestingly, no differences in PH were observed between the Northern and Southern sets. A clear separation between the sets indicates that there was a limited exchange of beneficial alleles and clear potential to improve Northern germplasm by applying marker assisted or genomic selection for the key adaptive traits.

In conclusion, we have identified a total of 18 consistent QTL for four major agronomic traits in a panel of 299 spring wheat genotypes tested over 12 trials. Four QTL were detected for thousand kernel weight, four QTL for GPC, and four QTL for PH. A total of six QTL were identified for earliness with three QTL associated with DH trait and remaining three QTL were revealed when earliness was calibrated for temperature. These QTL provide valuable basis for marker assisted selection in spring wheat breeding for future climates in the Nordic–Baltic region. It might be beneficial to introduce existing spring wheat genetic resources from lower latitudes into the breeding programs of the Nordic countries to improve GY performance under the changing climate.

## Data availability statement

The original contributions presented in this study are included in the **Supplementary Material**. Further inquiries can be directed to the corresponding author.

## Author contributions

AA: Writing – review & editing, Writing – original draft, Visualization, Investigation, Formal analysis, Data curation. AG: Writing – review & editing, Writing – original draft, Visualization, Resources, Investigation, Formal analysis, Data curation. RA: Writing – review & editing, Writing – original draft. IT: Writing – review & editing, Resources, Project administration, Investigation, Funding acquisition, Data curation. AI: Writing – review & editing, Resources, Investigation, Data curation. MB: Writing – review & editing, Resources, Project administration, Investigation, Funding acquisition, Data curation. VF: Writing – review & editing, Investigation. HK: Writing – review & editing, Project administration, Funding acquisition. TM: Writing – review & editing, Investigation, Data curation. ML: Writing – review & editing, Project administration, Investigation, Funding acquisition, Data curation. GB: Writing – review & editing, Writing – original draft, Supervision, Project administration, Investigation, Funding acquisition.
