# Supplementary material for: Genome-wide association study reveals 18 QTL for major agronomic traits in a Nordic–Baltic spring wheat germplasm
Source: Front Plant Sci. 2024 Jun 21;15:1393170. doi: 10.3389/fpls.2024.1393170 (PMC11224466; doi:10.3389/fpls.2024.1393170)
Supplement: Supplementary file 1 [file DataSheet_1.docx]

**Genome-wide association study reveals 18 QTL for major agronomic traits in a Nordic-Baltic spring wheat germplasm**

Andrius Aleliūnas1, Andrii Gorash 1, Rita Armonienė1, Ilmar Tamm2, Anne Ingver2, Māra Bleidere3, Valentīna Fetere3, Hannes Kollist4, Tomasz Mroz5, Morten Lillemo5, Gintaras Brazauskas1

**Supplementary material: Figures S1-S36**

**
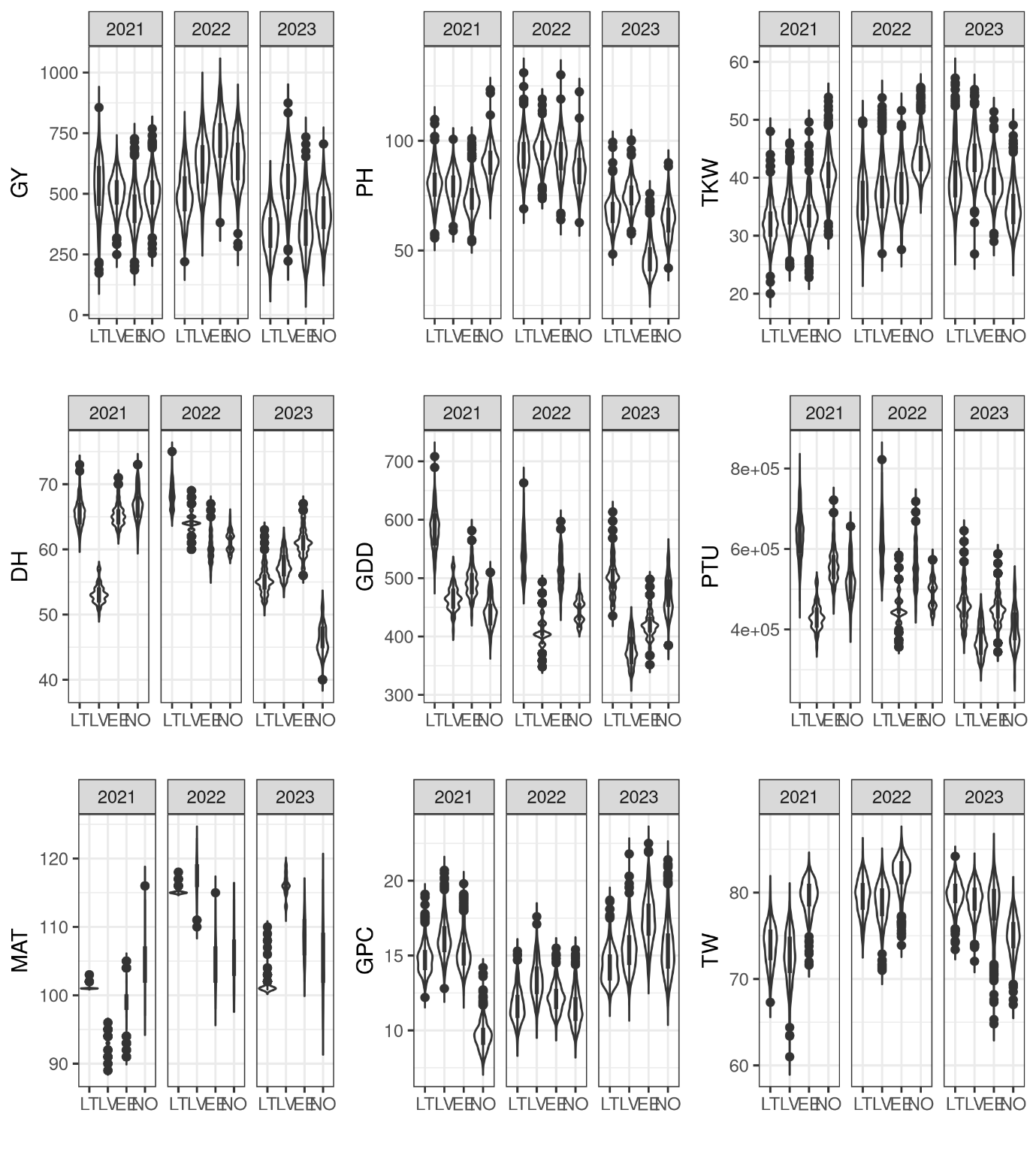
**

**Figure S1**. Violin plots showing the distribution of values for days to heading (DH), growing degree days (GDD) and photo thermal units (PTU) from sowing till heading, plant height (PH), thousand kernel weight (TKW), grain yield (GY), grain protein content (GPC), and test weight (TW) in four environments (LT, LV, EE, and NO) and in three growth seasons, 2021-2023.


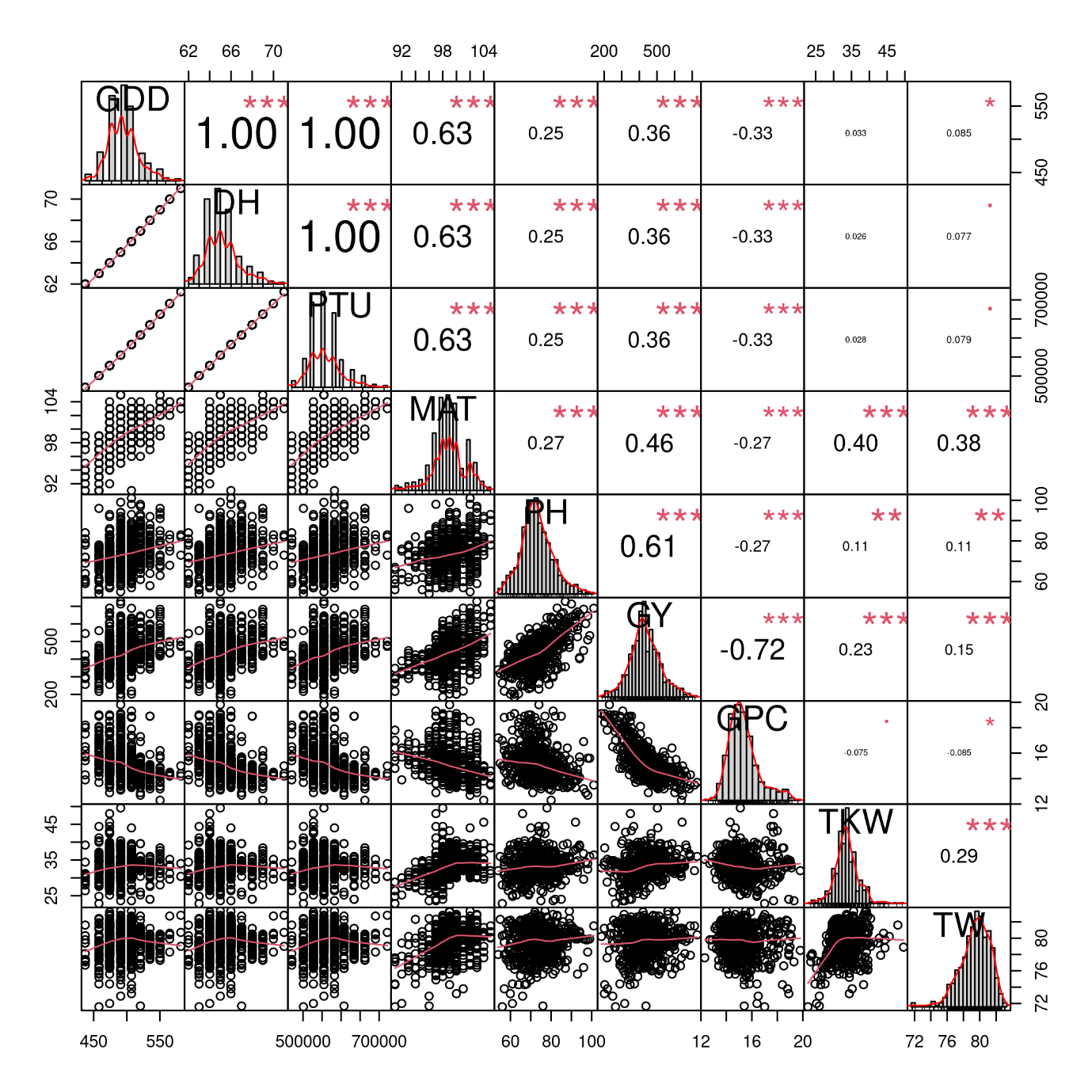


**Figure S2**. Correlation matrix between the traits using the data from Estonian 2021 trial.

All correlations were highly significant with the p-values < 0.001 as indicated by ***. Trait histograms are provided diagonally, while the scatterplots can be observed on the lower left part of the plot. GDD – growing degree days to heading, DH – heading date, PTU – photothermal units to heading, MAT – maturity date, PH – plant height, GY – grain yield, GPC – grain protein content, TKW – thousand kernel weight, TW – test weight. Symbols “***”, “**”, “*”, “.”, " “ represent p-values of 0, 0.001, 0.01, 0.05, 0.1, 1, respectively.


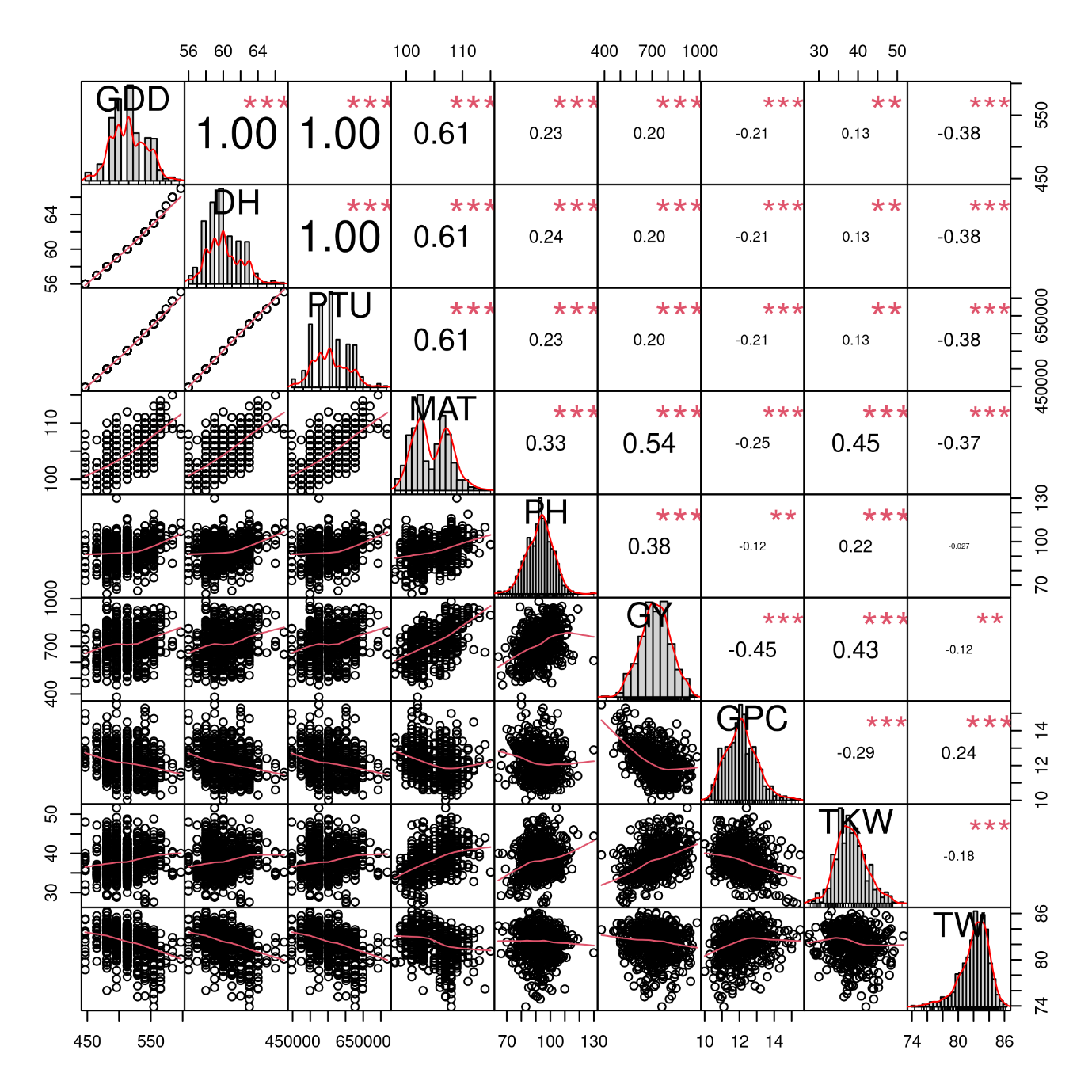


**Figure S3**. Correlation matrix between the traits using the data from Estonian 2022 trial.

All correlations were highly significant with the p-values < 0.001 as indicated by ***. Trait histograms are provided diagonally, while the scatterplots can be observed on the lower left part of the plot. GDD – growing degree days to heading, DH – heading date, PTU – photothermal units to heading, MAT – maturity date, PH – plant height, GY – grain yield, GPC – grain protein content, TKW – thousand kernel weight, TW – test weight. Symbols “***”, “**”, “*”, “.”, " “ represent p-values of 0, 0.001, 0.01, 0.05, 0.1, and 1, respectively.

**
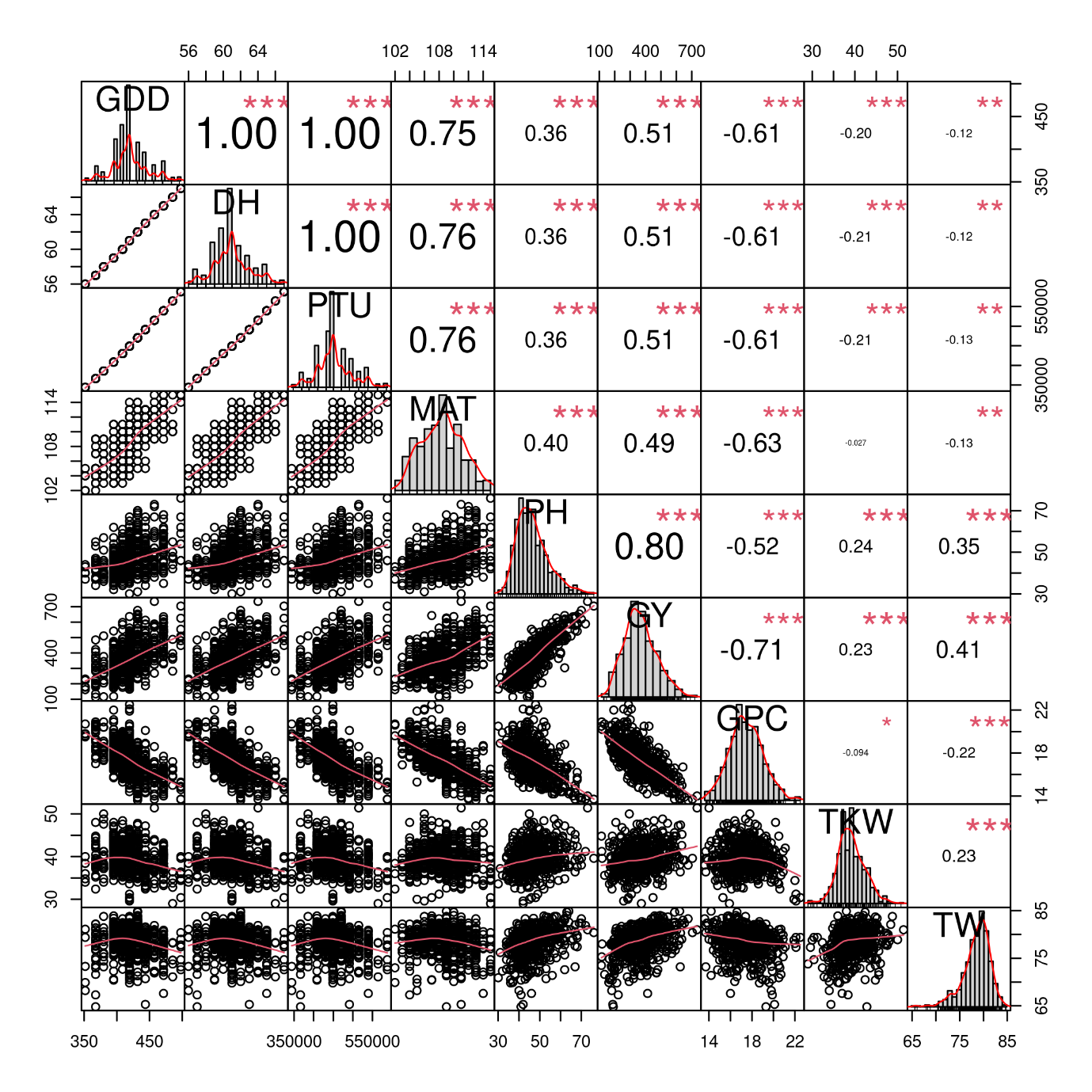
Figure S4**. Correlation matrix between the traits using the data from Estonian 2023 trial.

All correlations were highly significant with the p-values < 0.001 as indicated by ***. Trait histograms are provided diagonally, while the scatterplots can be observed on the lower left part of the plot. GDD – growing degree days to heading, DH – heading date, PTU – photothermal units to heading, MAT – maturity date, PH – plant height, GY – grain yield, GPC – grain protein content, TKW – thousand kernel weight, TW – test weight. Symbols “***”, “**”, “*”, “.”, " “ represent p-values of 0, 0.001, 0.01, 0.05, 0.1, 1, respectively.


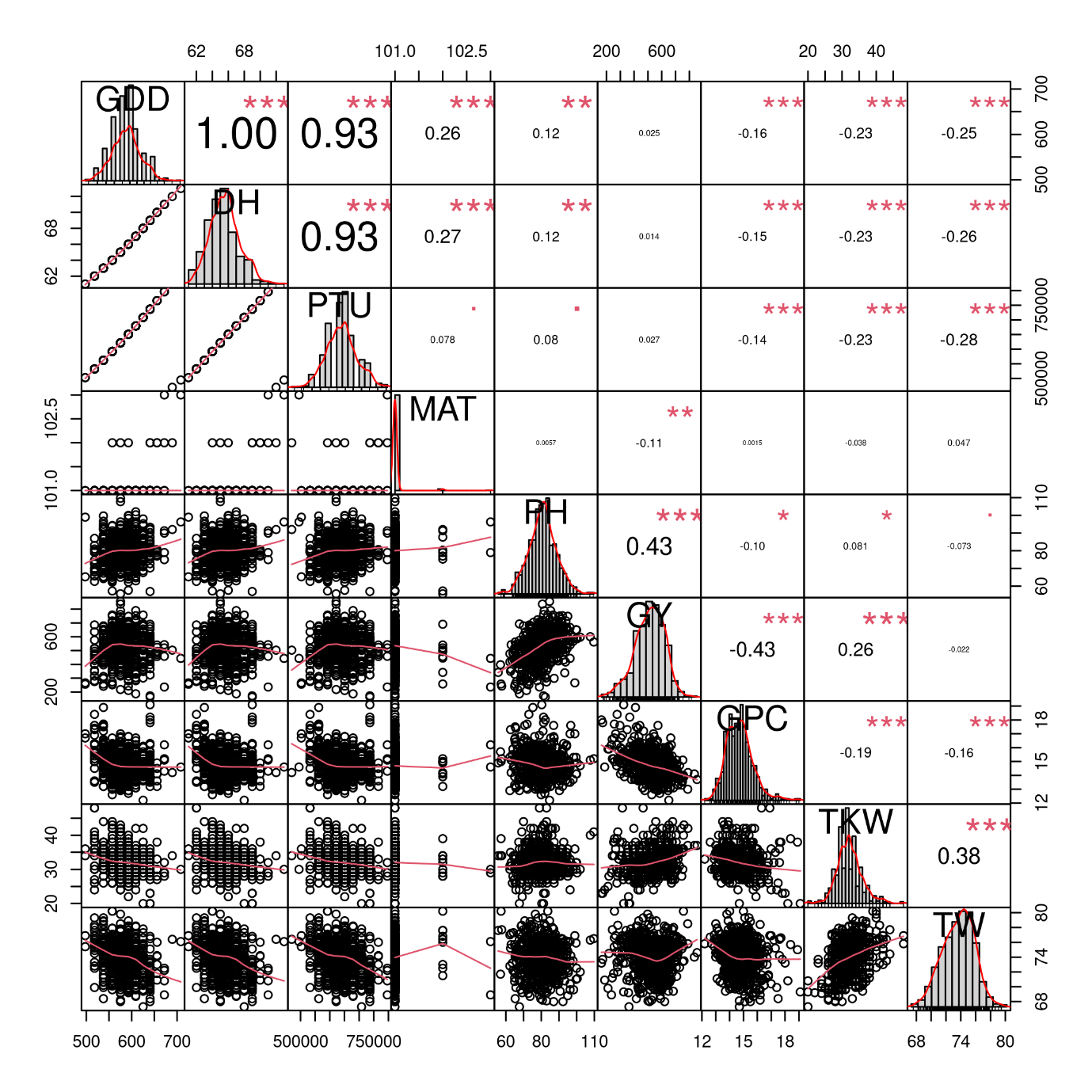


**Figure S5**. Correlation matrix between the traits using the data from Lithuanian 2021 trial.

All correlations were highly significant with the p-values < 0.001 as indicated by ***. Trait histograms are provided diagonally, while the scatterplots can be observed on the lower left part of the plot. GDD – growing degree days to heading, DH – heading date, PTU – photothermal units to heading, MAT – maturity date, PH – plant height, GY – grain yield, GPC – grain protein content, TKW – thousand kernel weight, TW – test weight. Symbols “***”, “**”, “*”, “.”, " “ represent p-values of 0, 0.001, 0.01, 0.05, 0.1, 1, respectively.


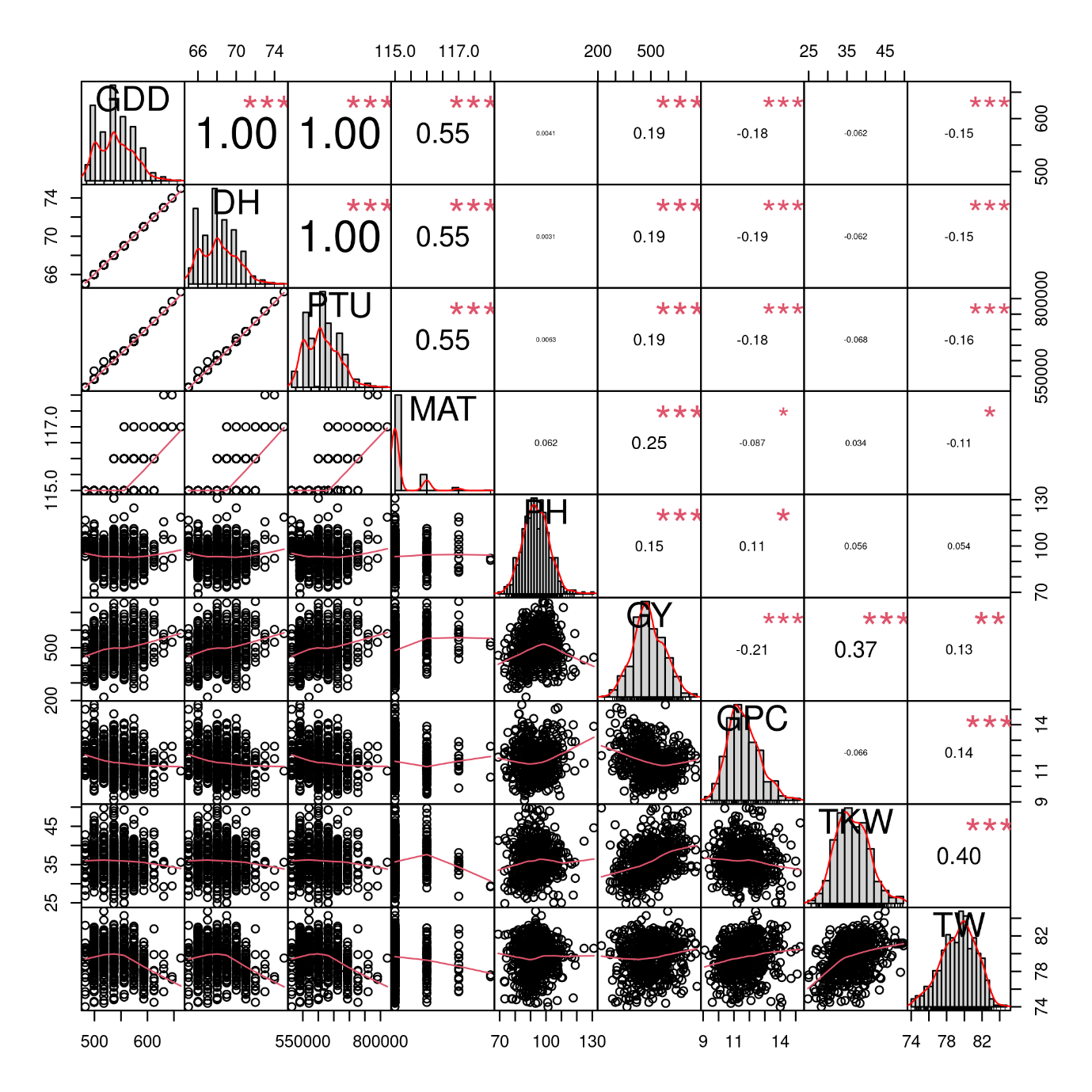


**Figure S6**. Correlation matrix between the traits using the data from Lithuanian 2022 trial.

All correlations were highly significant with the p-values < 0.001 as indicated by ***. Trait histograms are provided diagonally, while the scatterplots can be observed on the lower left part of the plot. GDD – growing degree days to heading, DH – heading date, PTU – photothermal units to heading, MAT – maturity date, PH – plant height, GY – grain yield, GPC – grain protein content, TKW – thousand kernel weight, TW – test weight. Symbols “***”, “**”, “*”, “.”, " “ represent p-values of 0, 0.001, 0.01, 0.05, 0.1, 1, respectively.

**
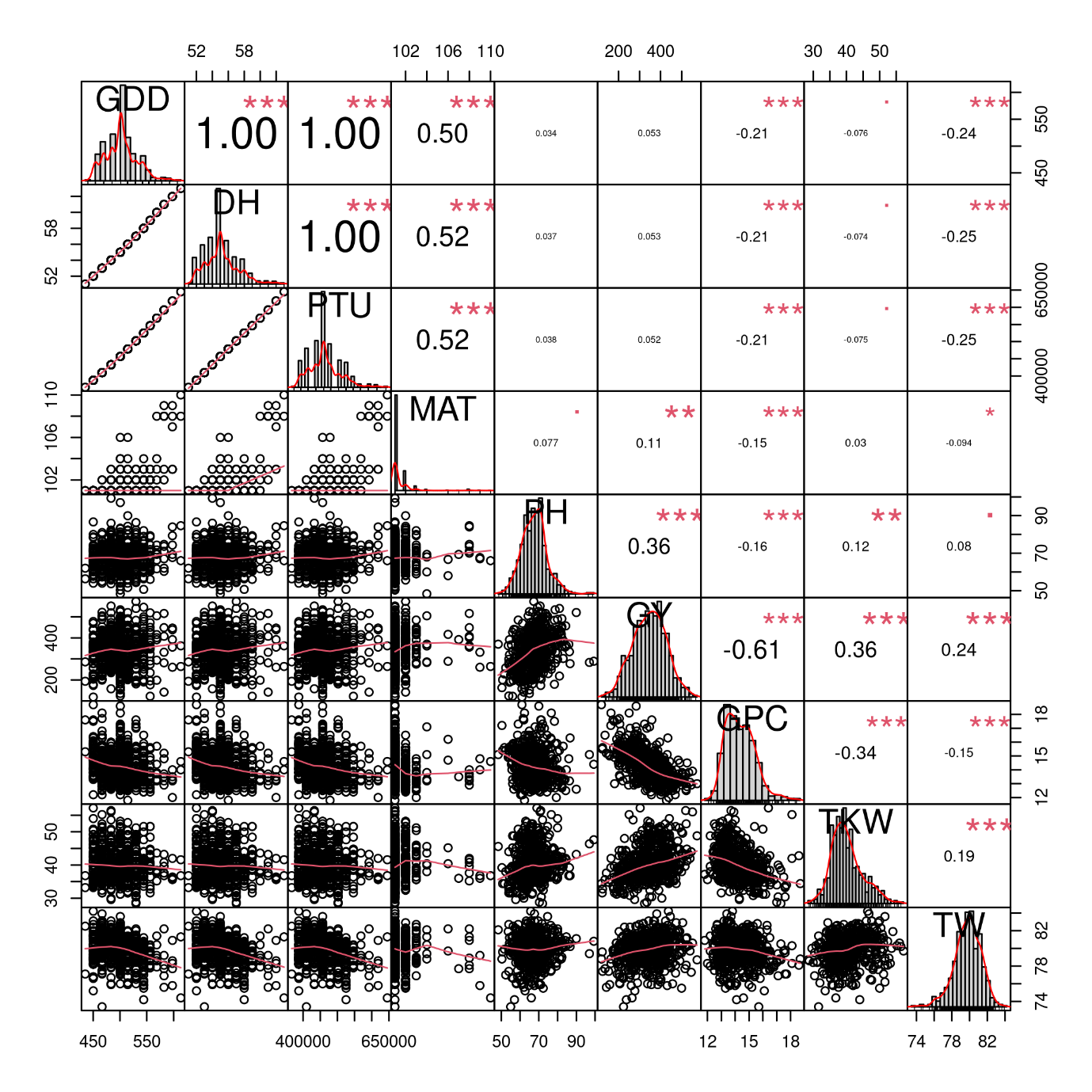
Figure S7**. Correlation matrix between the traits using the data from Lithuanian 2023 trial.

All correlations were highly significant with the p-values < 0.001 as indicated by ***. Trait histograms are provided diagonally, while the scatterplots can be observed on the lower left part of the plot. GDD – growing degree days to heading, DH – heading date, PTU – photothermal units to heading, MAT – maturity date, PH – plant height, GY – grain yield, GPC – grain protein content, TKW – thousand kernel weight, TW – test weight. Symbols “***”, “**”, “*”, “.”, " “ represent p-values of 0, 0.001, 0.01, 0.05, 0.1, 1, respectively.


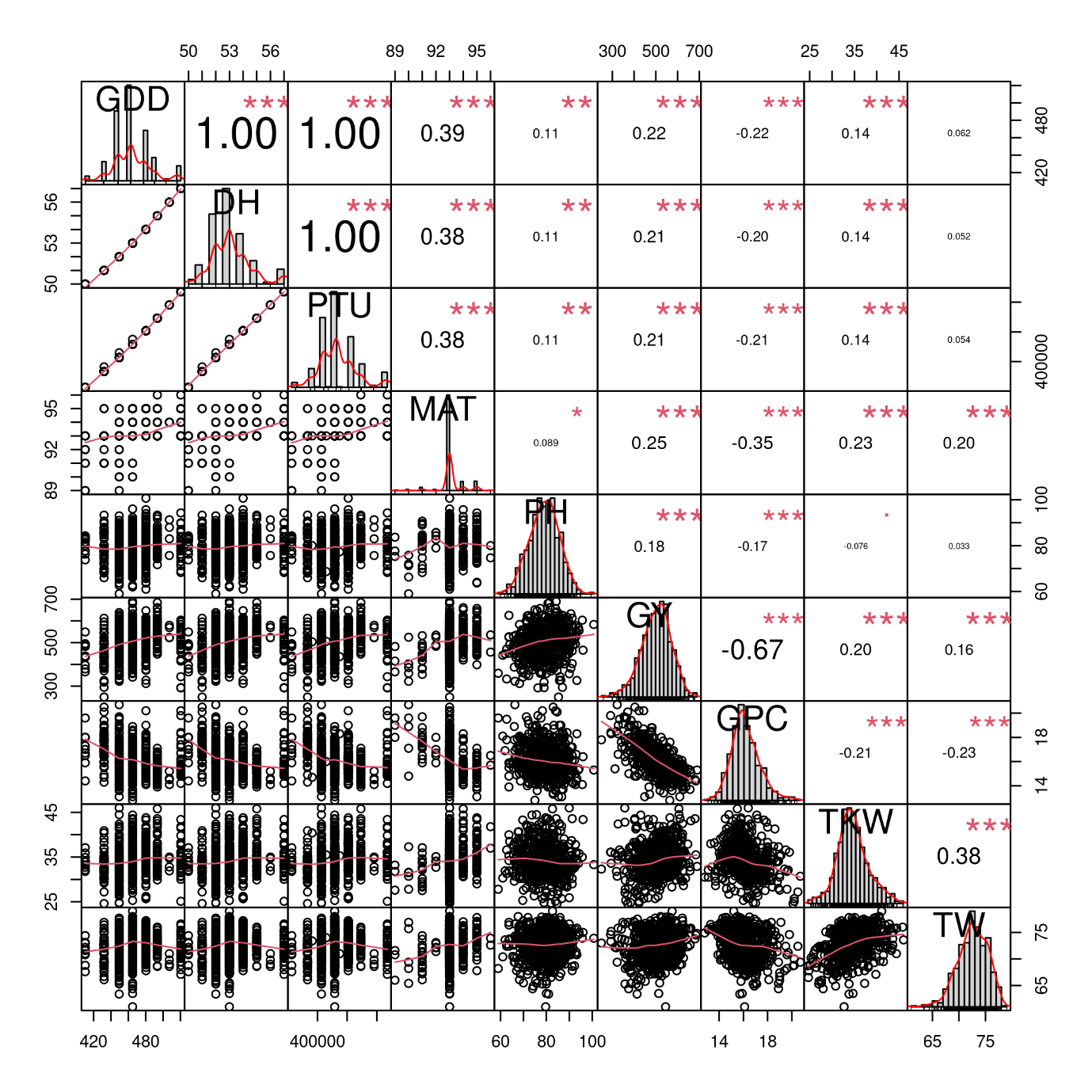


**Figure S8**. Correlation matrix between the traits using the data from Latvian 2021 trial.

All correlations were highly significant with the p-values < 0.001 as indicated by ***. Trait histograms are provided diagonally, while the scatterplots can be observed on the lower left part of the plot. GDD – growing degree days to heading, DH – heading date, PTU – photothermal units to heading, MAT – maturity date, PH – plant height, GY – grain yield, GPC – grain protein content, TKW – thousand kernel weight, TW – test weight. Symbols “***”, “**”, “*”, “.”, " “ represent p-values of 0, 0.001, 0.01, 0.05, 0.1, 1, respectively.


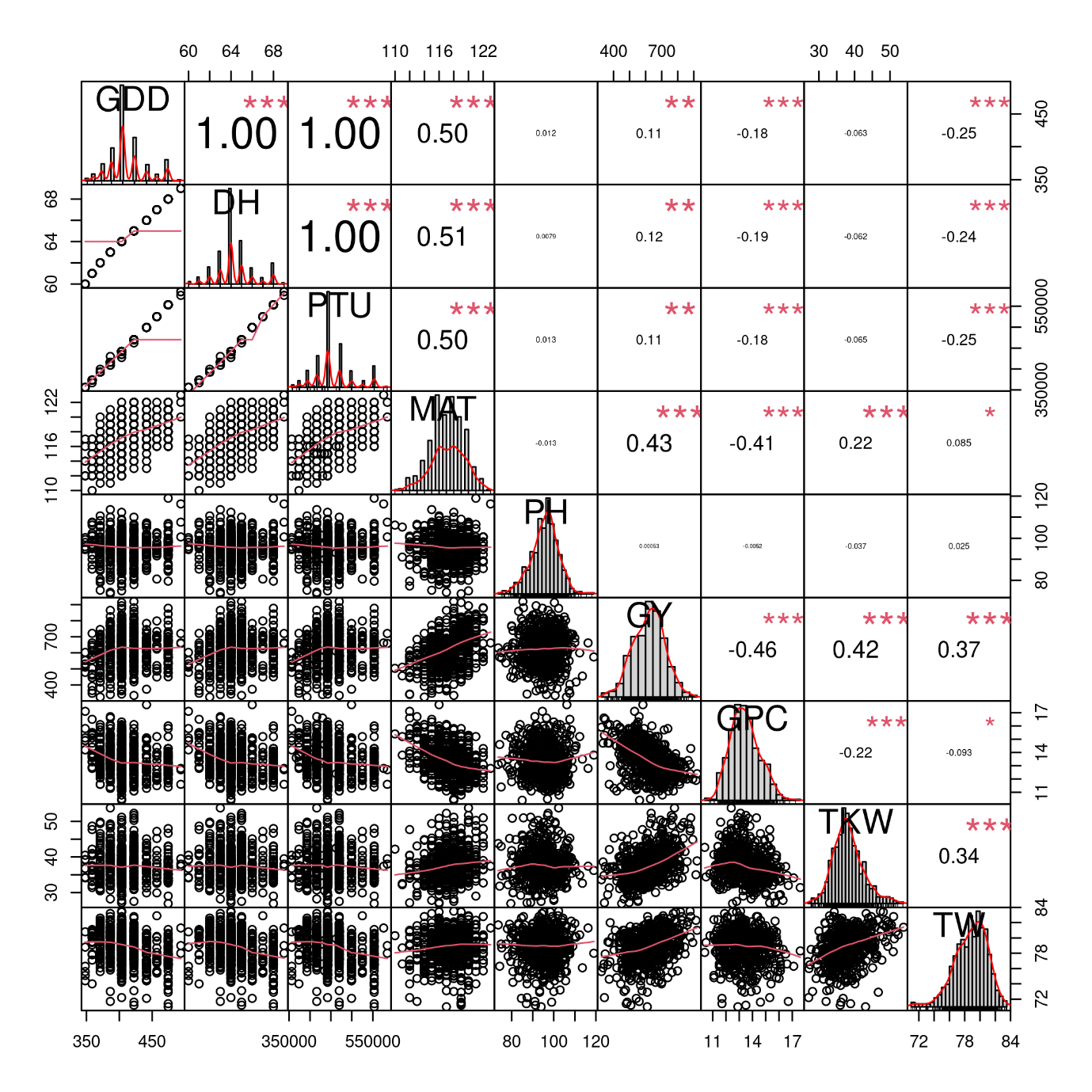


**Figure S9**. Correlation matrix between the traits using the data from Latvian 2022 trial.

All correlations were highly significant with the p-values < 0.001 as indicated by ***. Trait histograms are provided diagonally, while the scatterplots can be observed on the lower left part of the plot. GDD – growing degree days to heading, DH – heading date, PTU – photothermal units to heading, MAT – maturity date, PH – plant height, GY – grain yield, GPC – grain protein content, TKW – thousand kernel weight, TW – test weight. Symbols “***”, “**”, “*”, “.”, " “ represent p-values of 0, 0.001, 0.01, 0.05, 0.1, 1, respectively.


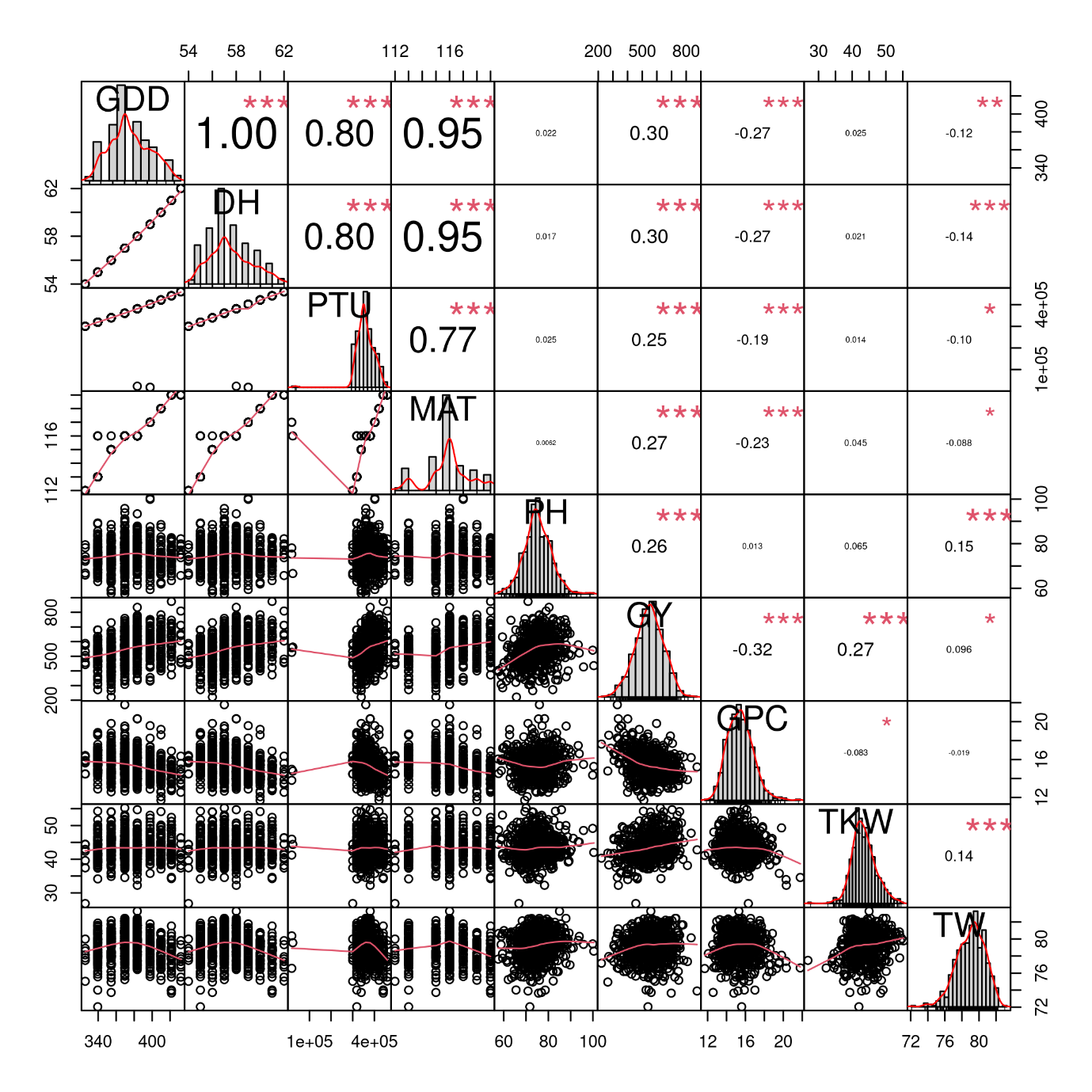


**Figure S10**. Correlation matrix between the traits using the data from Latvian 2023 trial.

All correlations were highly significant with the p-values < 0.001 as indicated by ***. Trait histograms are provided diagonally, while the scatterplots can be observed on the lower left part of the plot. GDD – growing degree days to heading, DH – heading date, PTU – photothermal units to heading, MAT – maturity date, PH – plant height, GY – grain yield, GPC – grain protein content, TKW – thousand kernel weight, TW – test weight. Symbols “***”, “**”, “*”, “.”, " “ represent p-values of 0, 0.001, 0.01, 0.05, 0.1, 1, respectively.


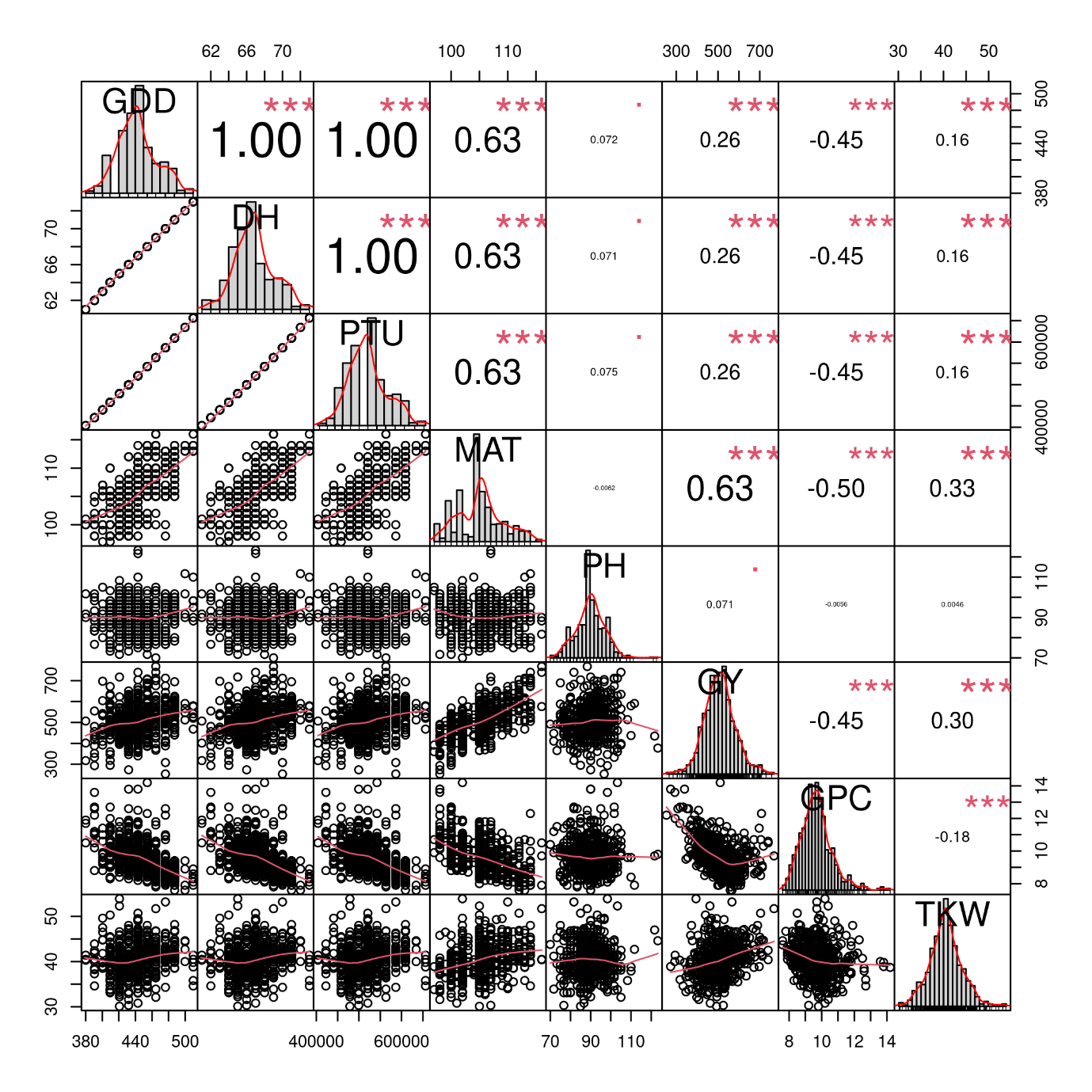


**Figure S11**. Correlation matrix between the traits using the data from Norwegian 2021 trial.

All correlations were highly significant with the p-values < 0.001 as indicated by ***. Trait histograms are provided diagonally, while the scatterplots can be observed on the lower left part of the plot. GDD – growing degree days to heading, DH – heading date, PTU – photothermal units to heading, MAT – maturity date, PH – plant height, GY – grain yield, GPC – grain protein content, TKW – thousand kernel weight, TW – test weight. Symbols “***”, “**”, “*”, “.”, " “ represent p-values of 0, 0.001, 0.01, 0.05, 0.1, 1, respectively.


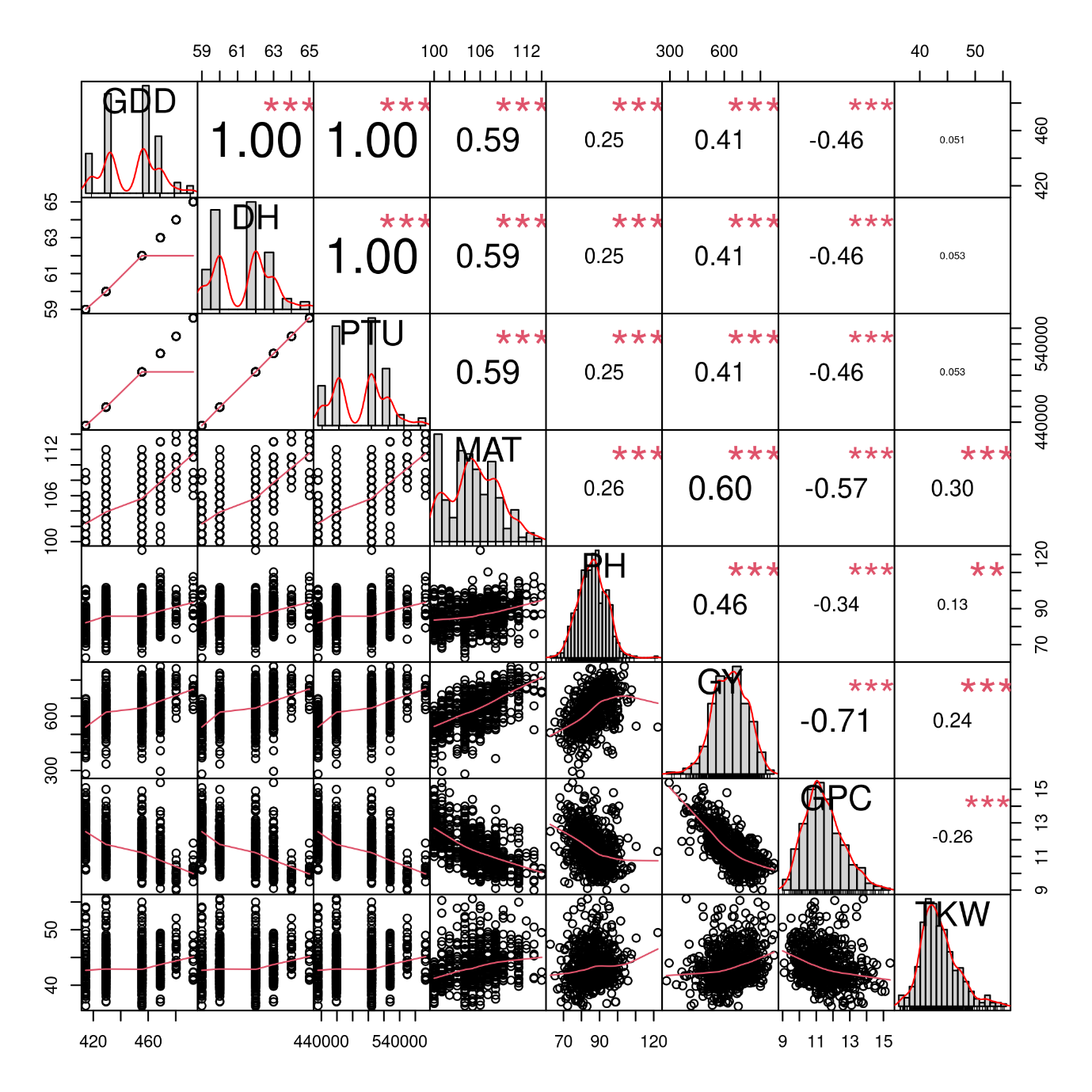


**Figure S12**. Correlation matrix between the traits using the data from Norwegian 2022 trial.

All correlations were highly significant with the p-values < 0.001 as indicated by ***. Trait histograms are provided diagonally, while the scatterplots can be observed on the lower left part of the plot. GDD – growing degree days to heading, DH – heading date, PTU – photothermal units to heading, MAT – maturity date, PH – plant height, GY – grain yield, GPC – grain protein content, TKW – thousand kernel weight, TW – test weight. Symbols “***”, “**”, “*”, “.”, " “ represent p-values of 0, 0.001, 0.01, 0.05, 0.1, 1, respectively.


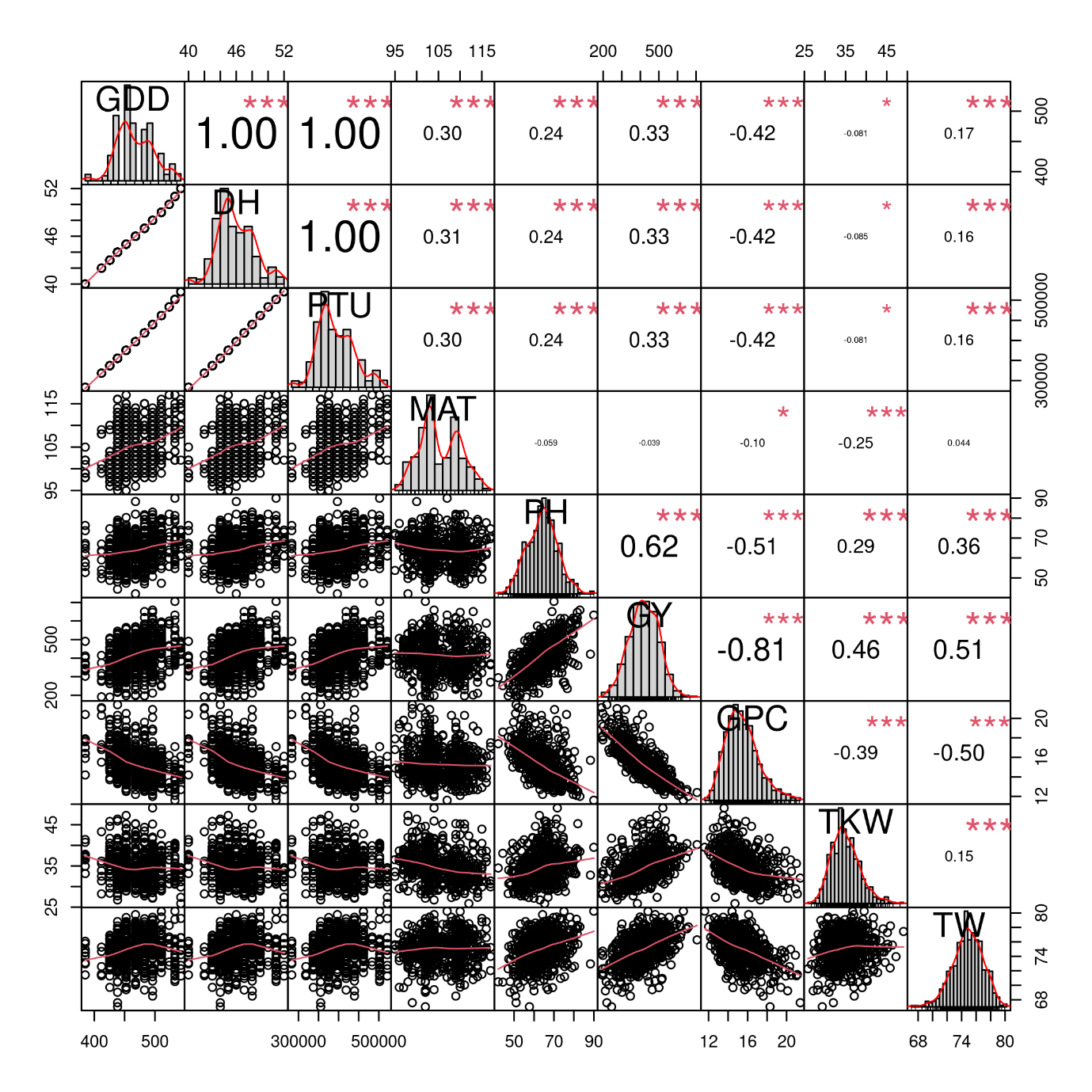


**Figure S13**. Correlation matrix between the traits using the data from Norwegian 2023 trial.

All correlations were highly significant with the p-values < 0.001 as indicated by ***. Trait histograms are provided diagonally, while the scatterplots can be observed on the lower left part of the plot. GDD – growing degree days to heading, DH – heading date, PTU – photothermal units to heading, MAT – maturity date, PH – plant height, GY – grain yield, GPC – grain protein content, TKW – thousand kernel weight, TW – test weight. Symbols “***”, “**”, “*”, “.”, " “ represent p-values of 0, 0.001, 0.01, 0.05, 0.1, 1, respectively.


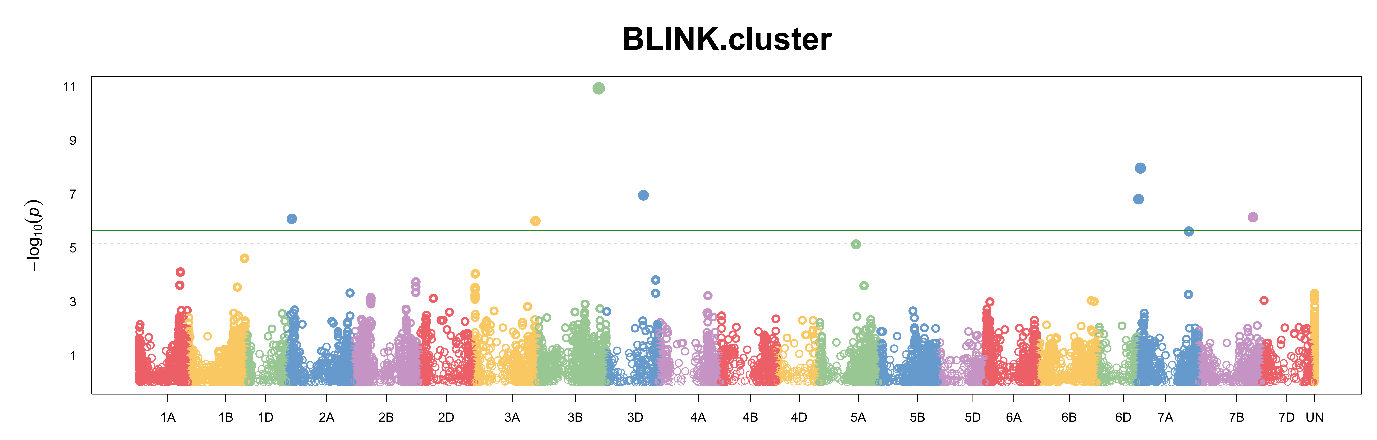


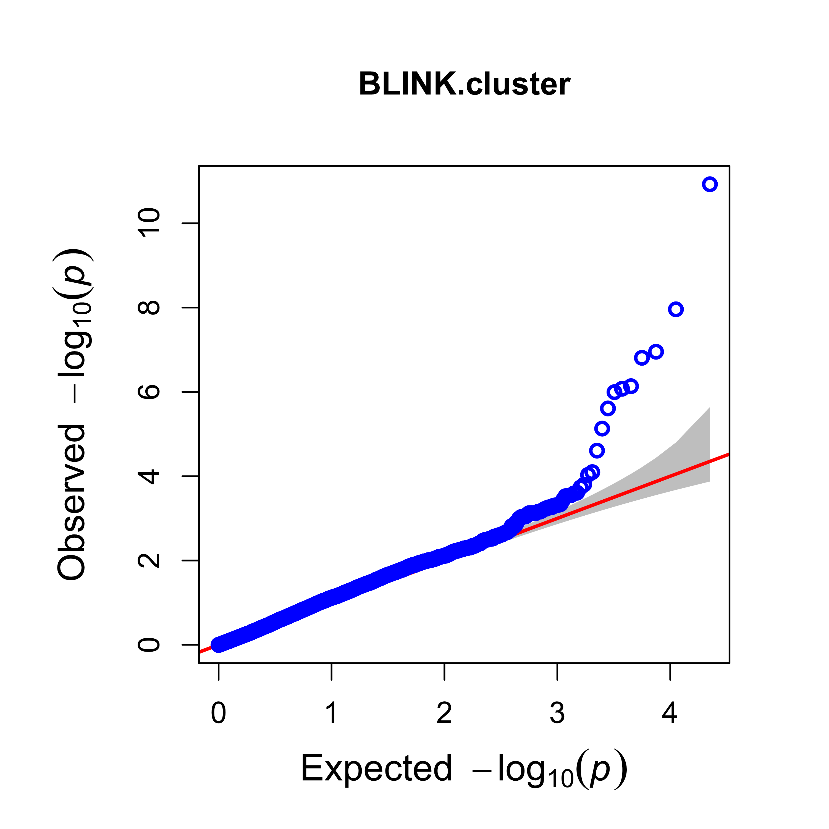


**Figure S14**. Manhattan and Q-Q plots of genome wide association study to identify genetic loci associated with segregation of Northern and Southern genotype clusters.


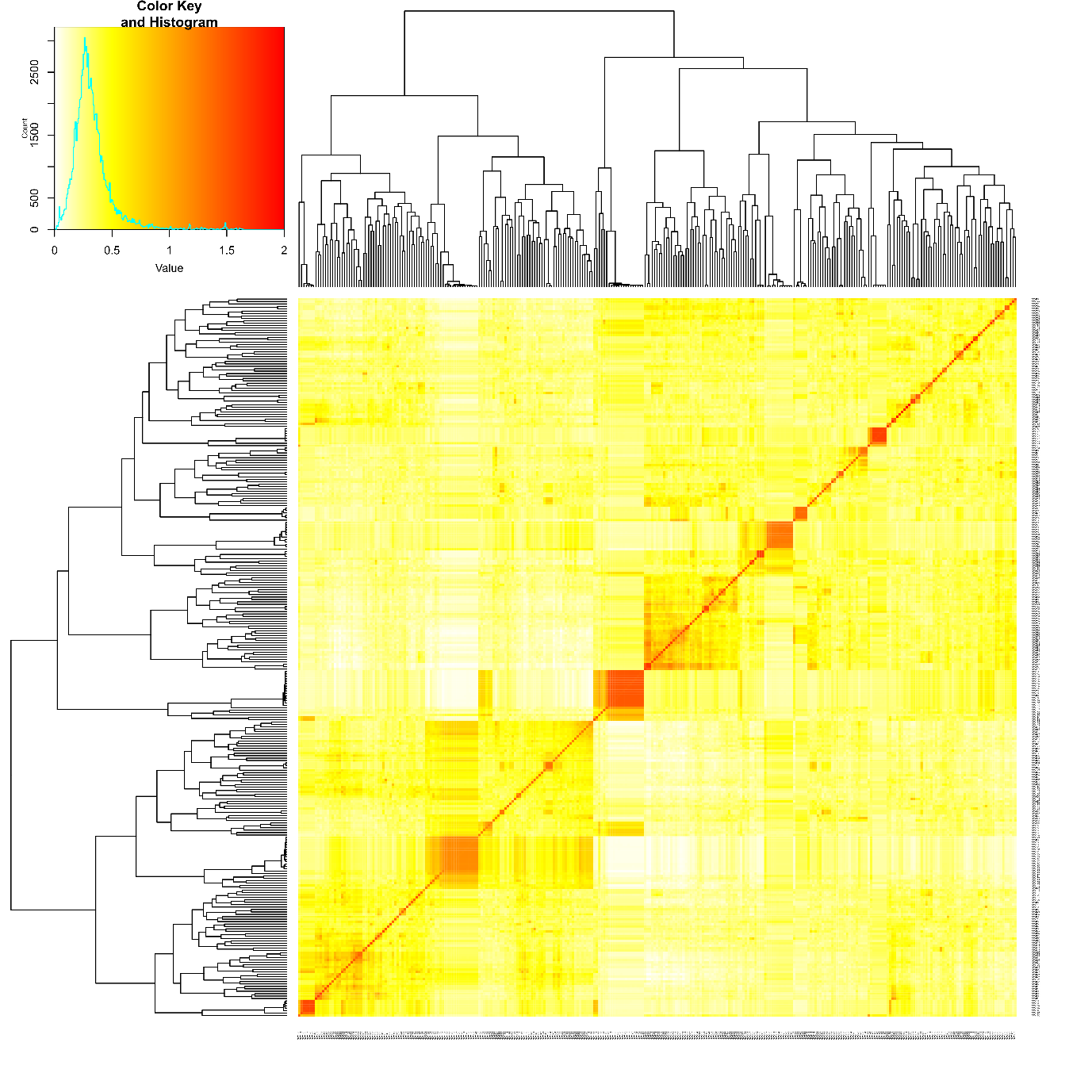


**Figure S15**. Kinship heatplot of NOBALwheat spring wheat association panel.


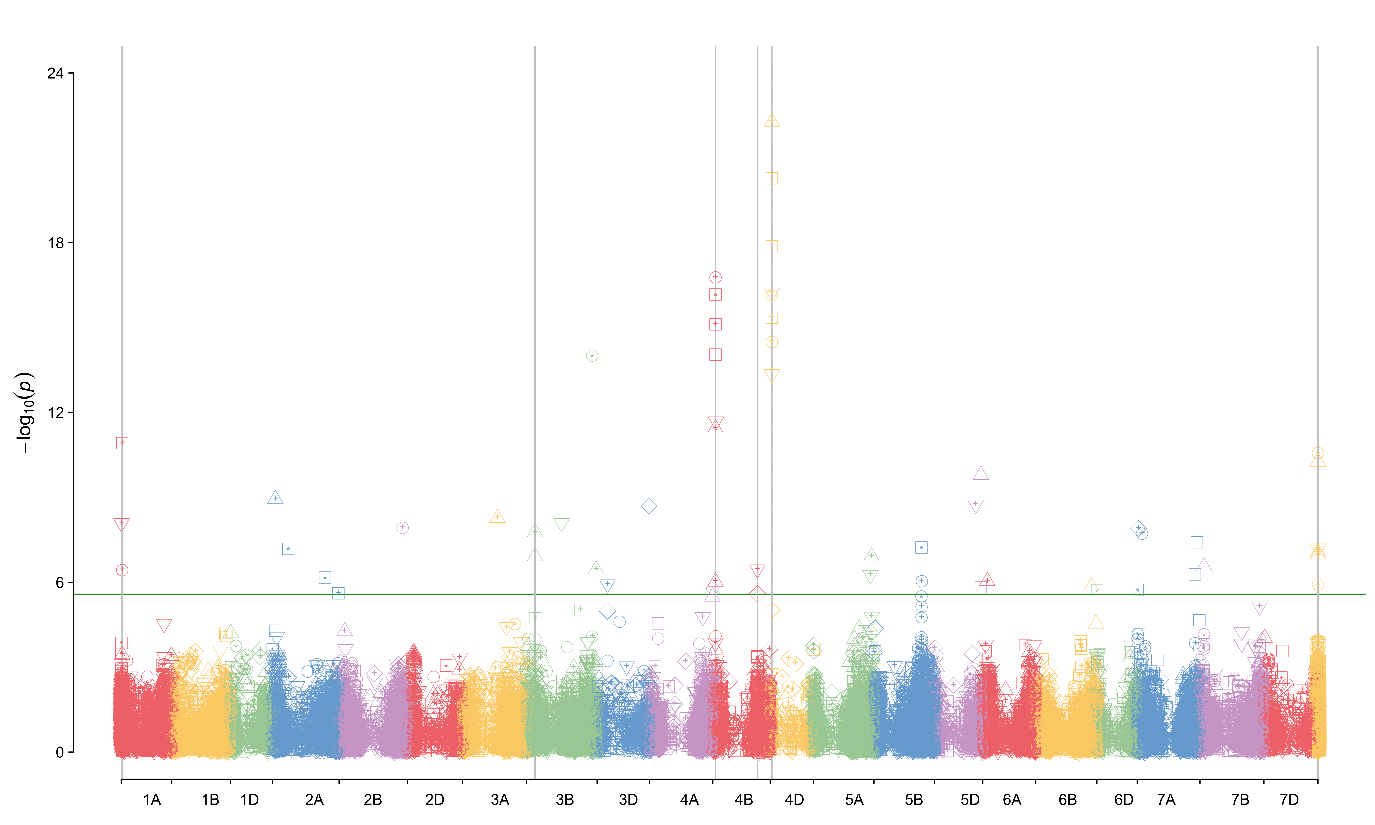
**Figure S16.** Manhattan plot for plant height (PH). The symbols define different environments.


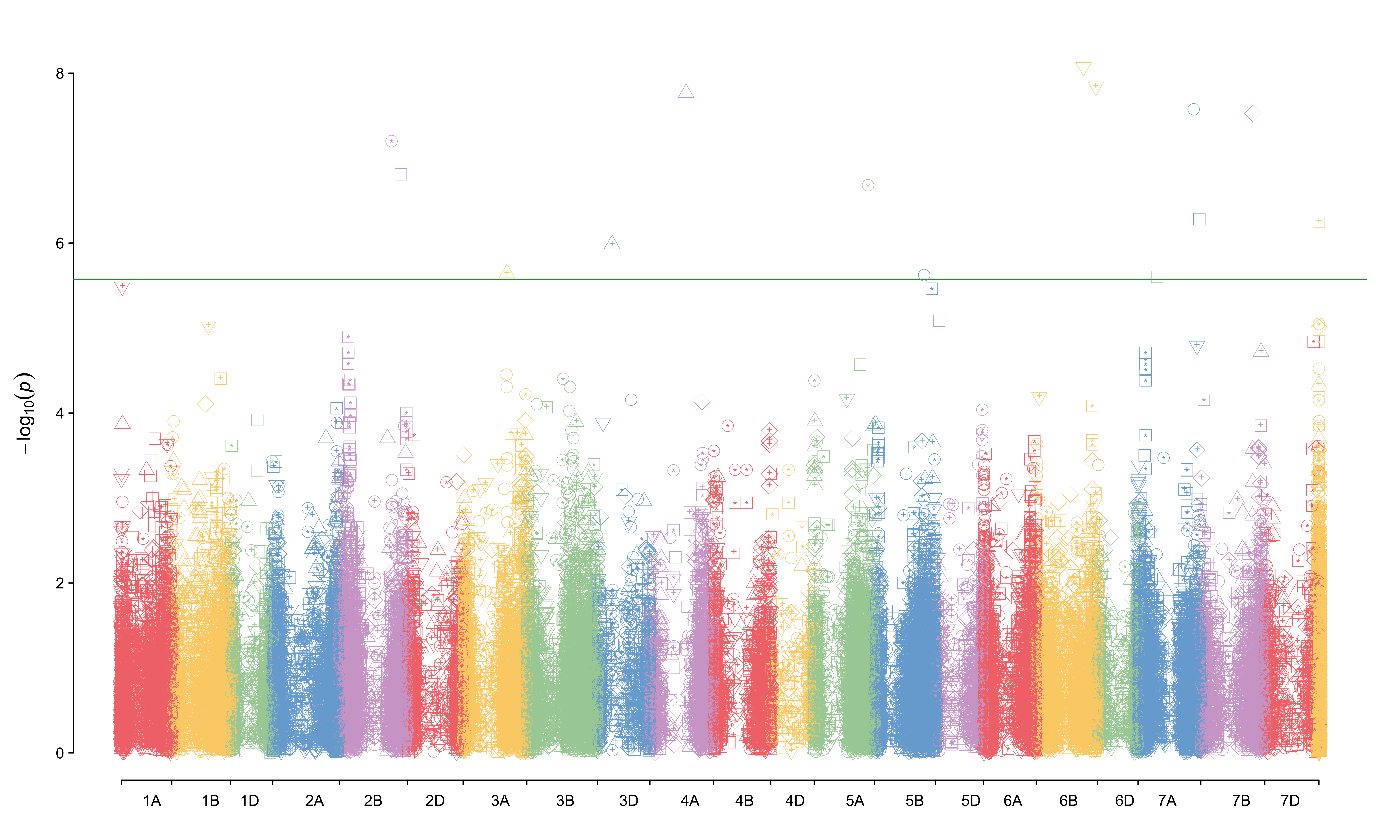
 **Figure S17.** Manhattan plot for grain yield (GY). The symbols define different environments.


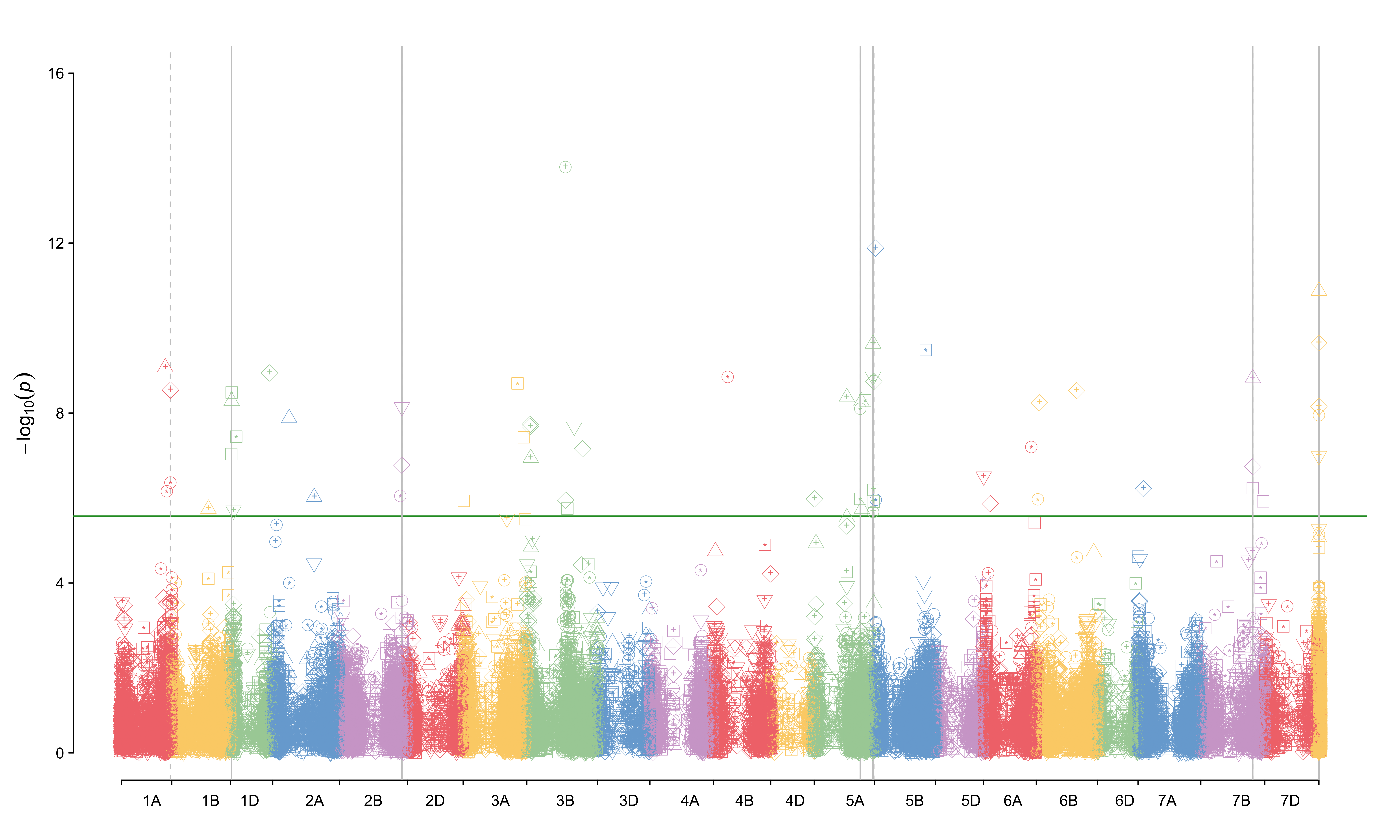
 **Figure S18.** Manhattan plot for grain protein content (GPC). The symbols define different environments.


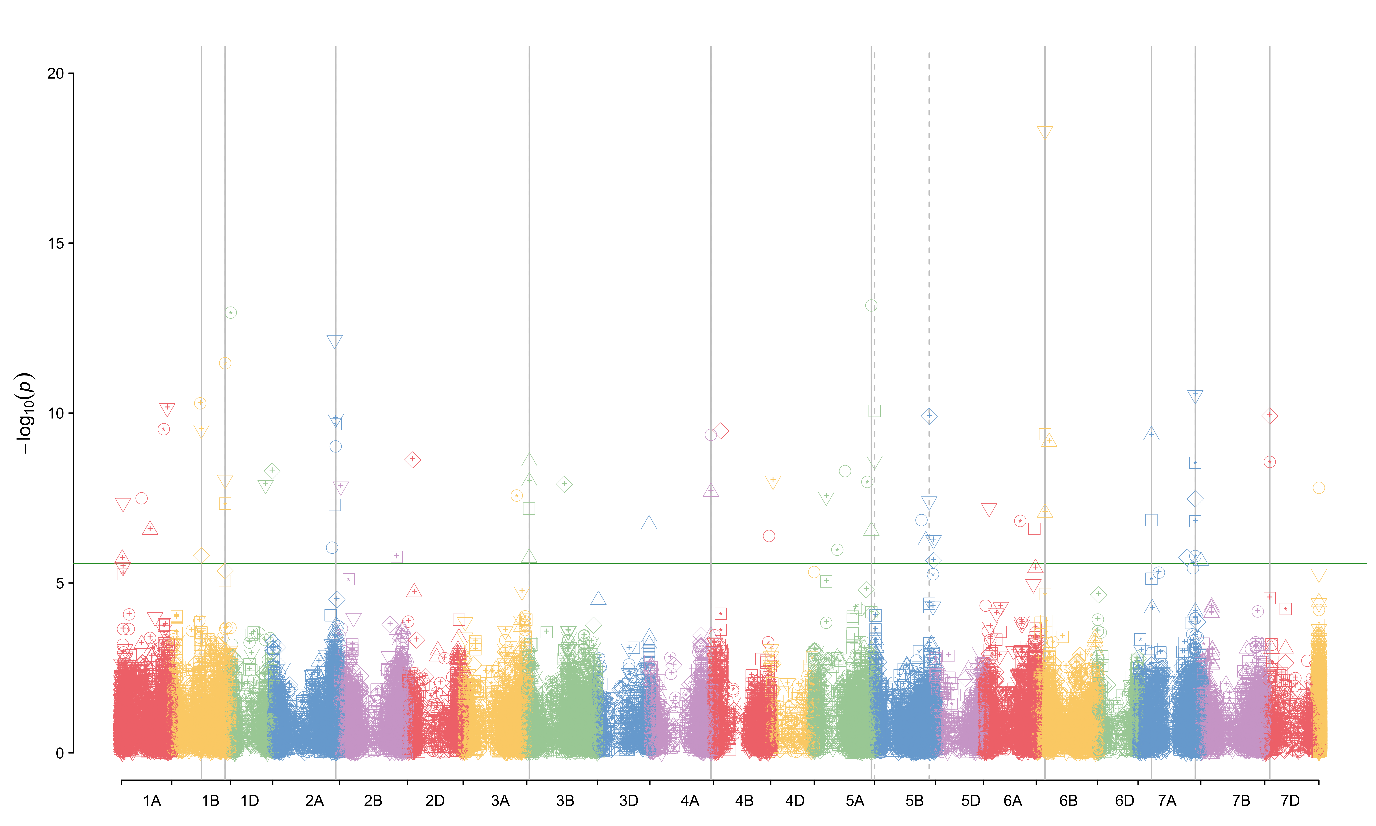
 **Figure S19.** Manhattan plot for thousand kernel weight (TKW). The symbols define different environments.


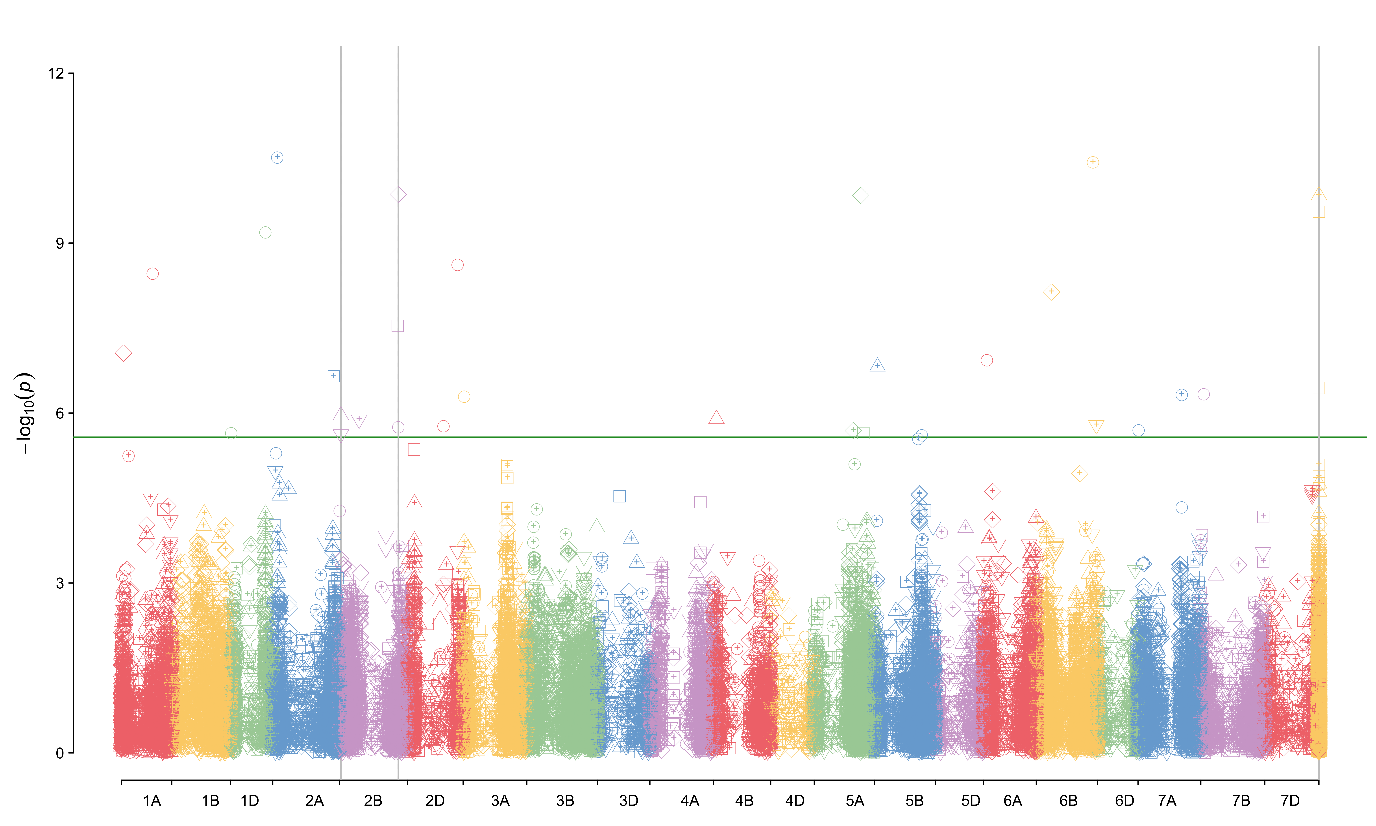
 **Figure S20.** Manhattan plot for grain test weight (TW). The symbols define different environments.


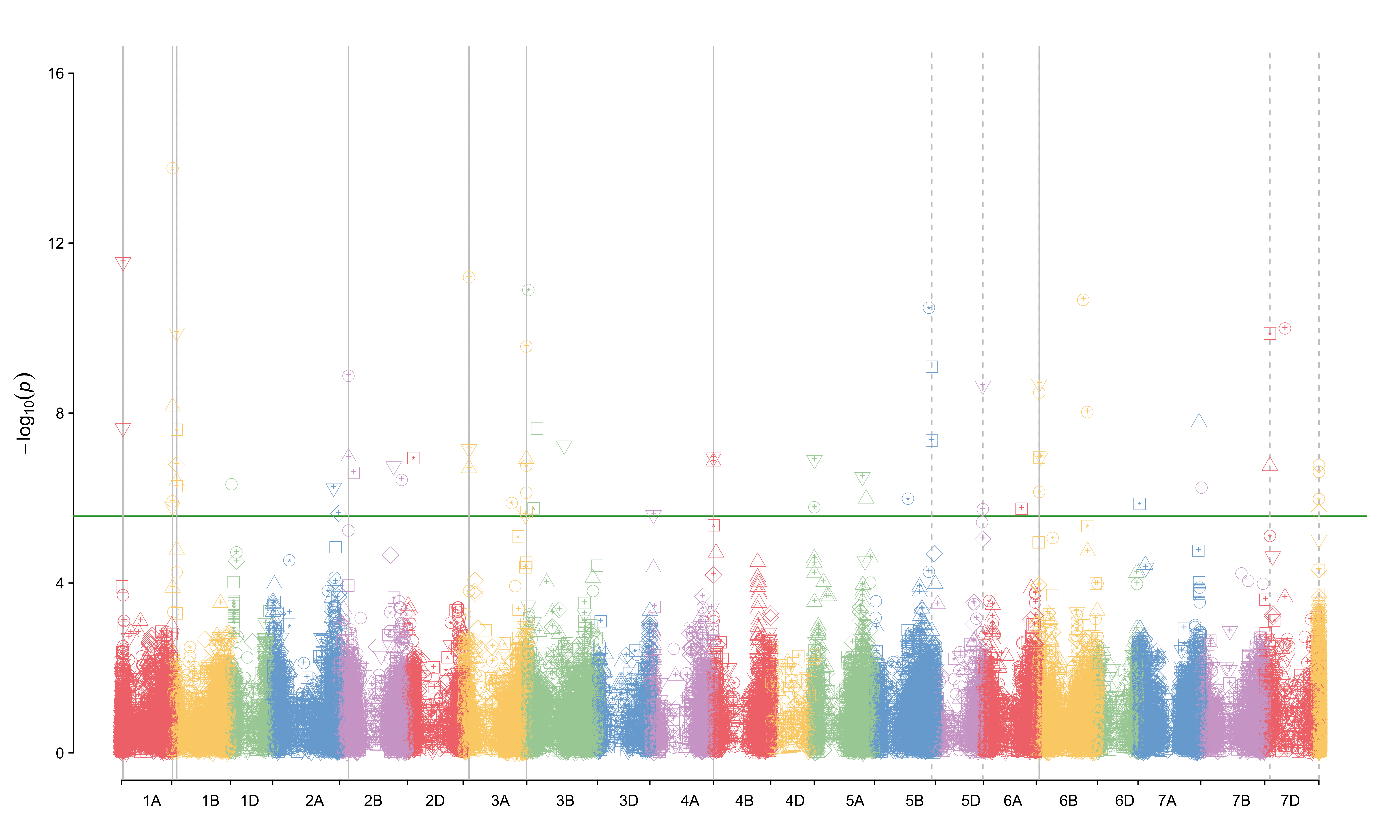


**Figure S21.** Manhattan plot for heading date (DH). The symbols define different environments.


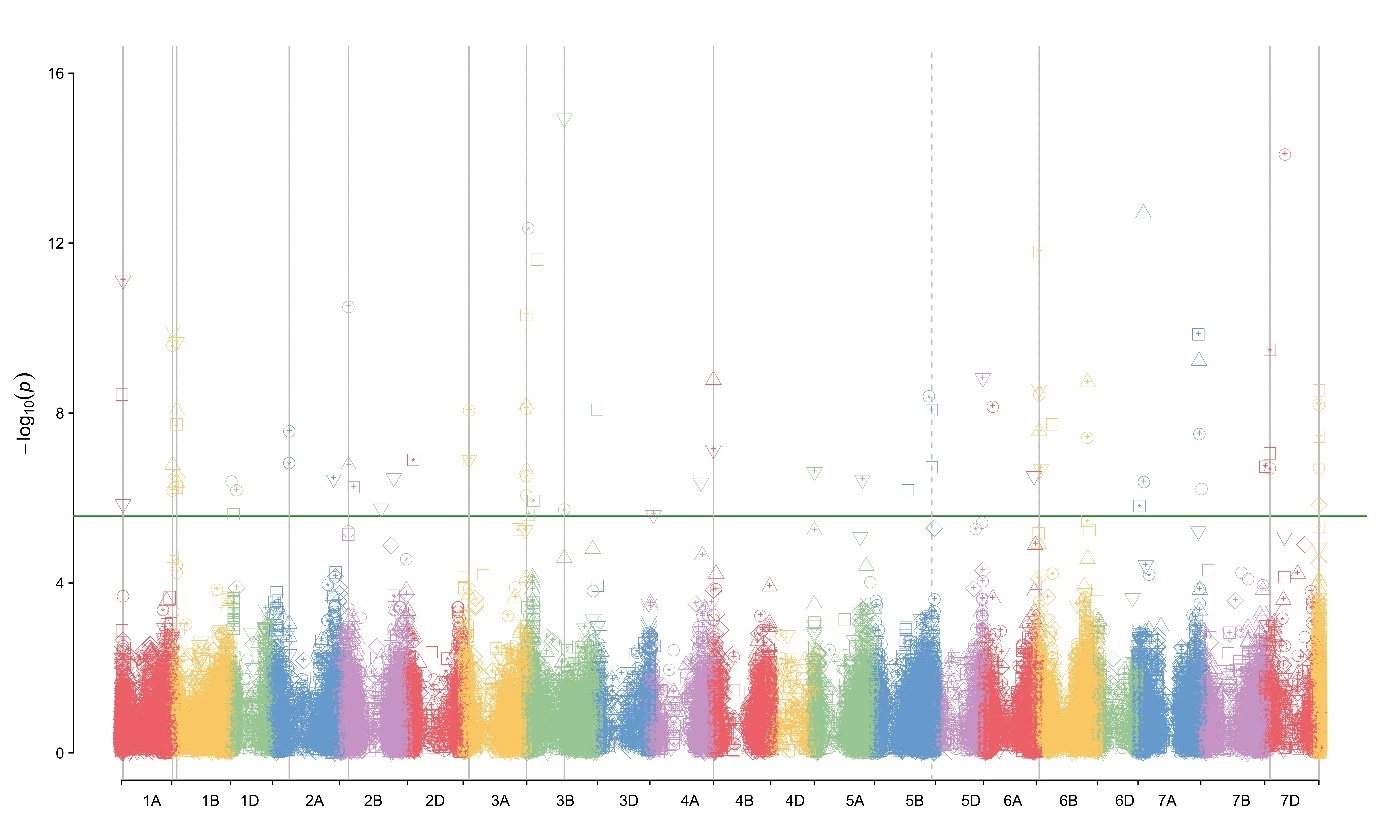


**Figure S22**. Manhattan plot for growing degree days to heading (GDD). The symbols define different environments.


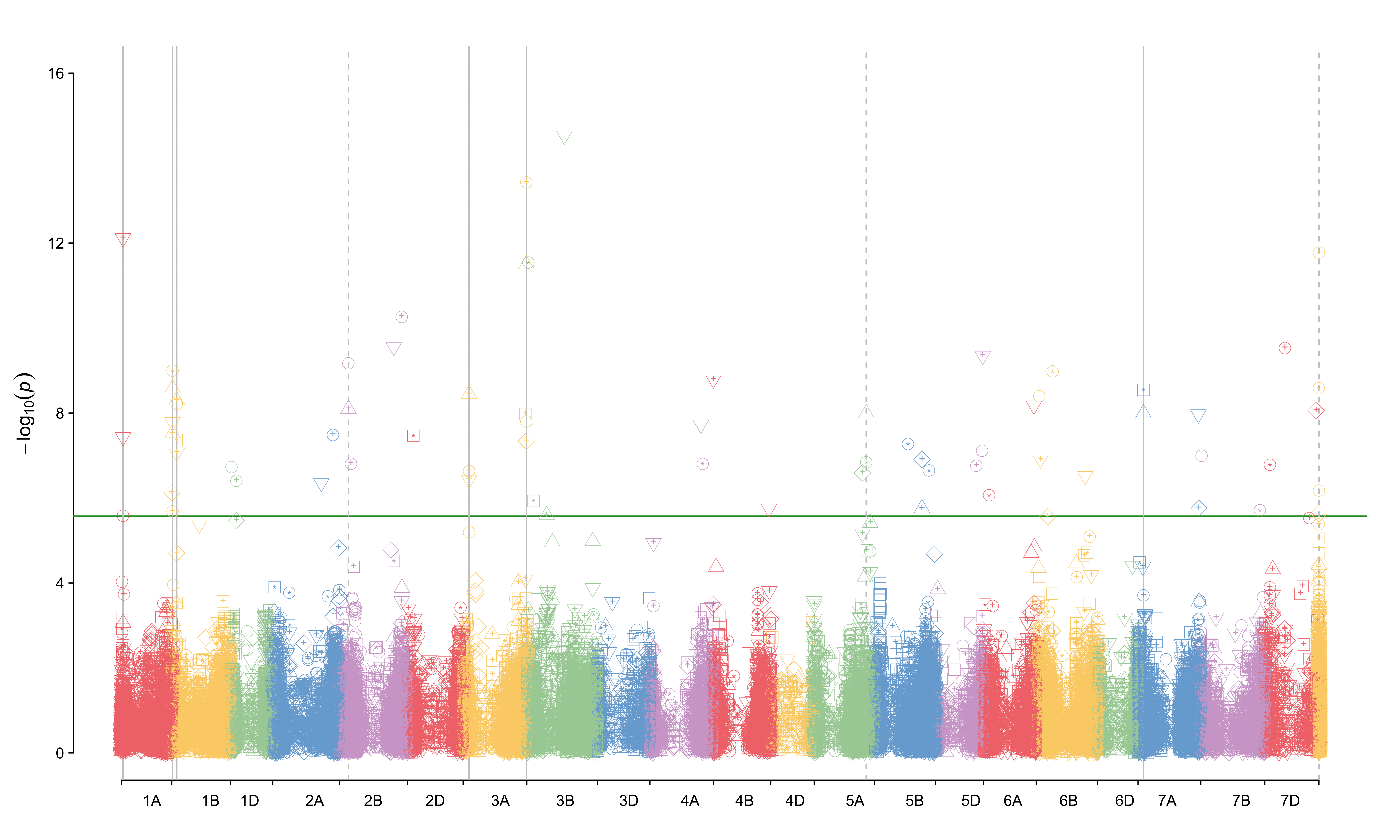


**Figure S23.** Manhattan plot for photothermal units to heading (PTU). The symbols define different environments.


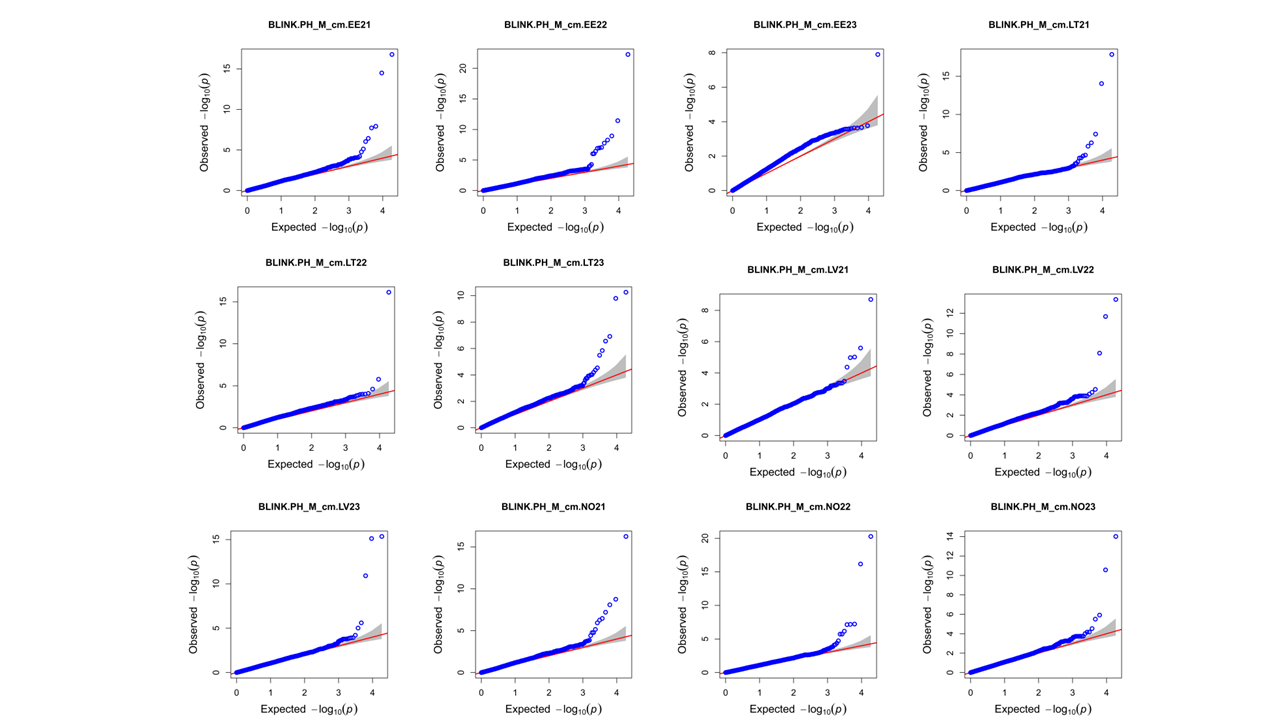


**Figure S24**. Q-Q plots for plant height (PH).


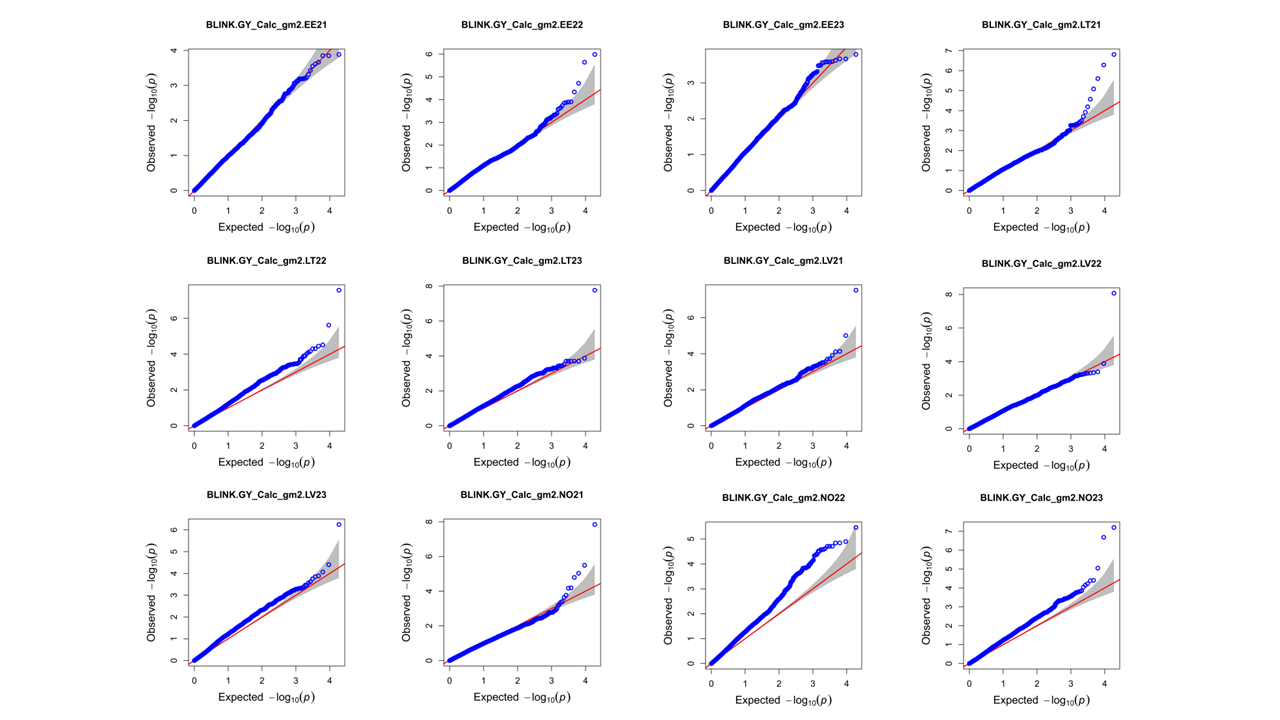


**Figure S25**. Q-Q plots for grain yield (GY).


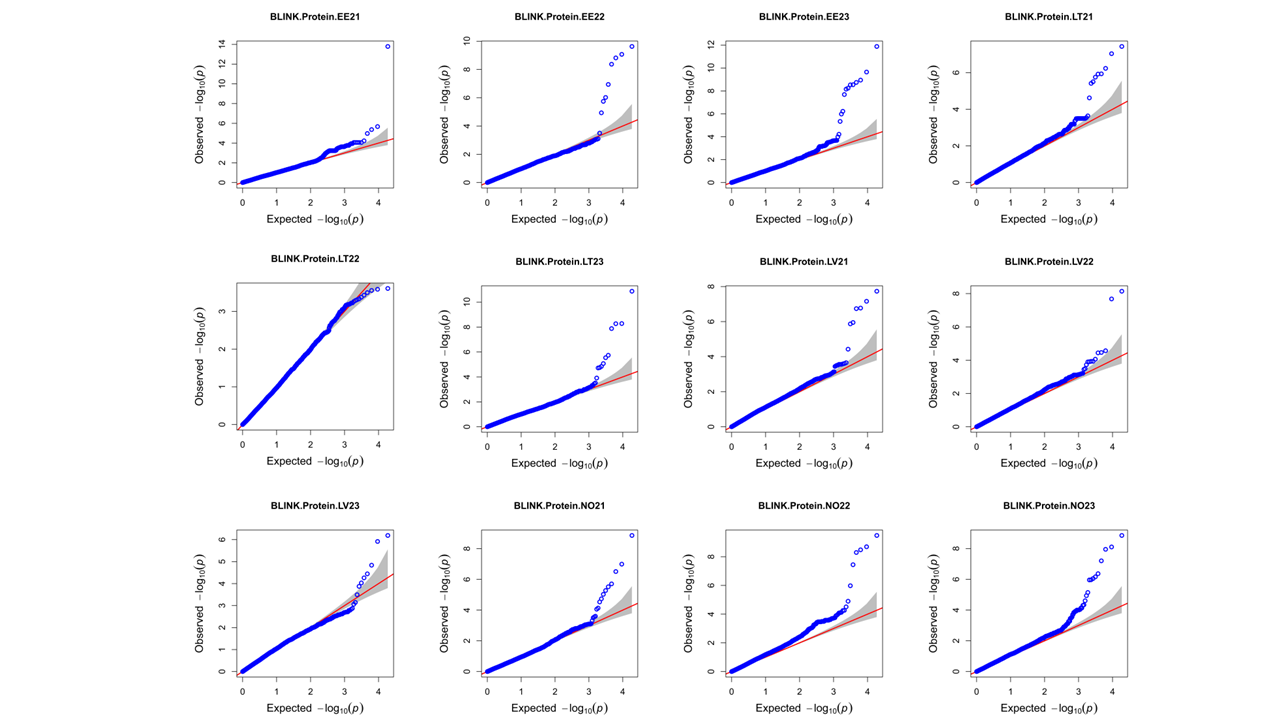


**Figure S26**. Q-Q plots for grain protein content (GPC).


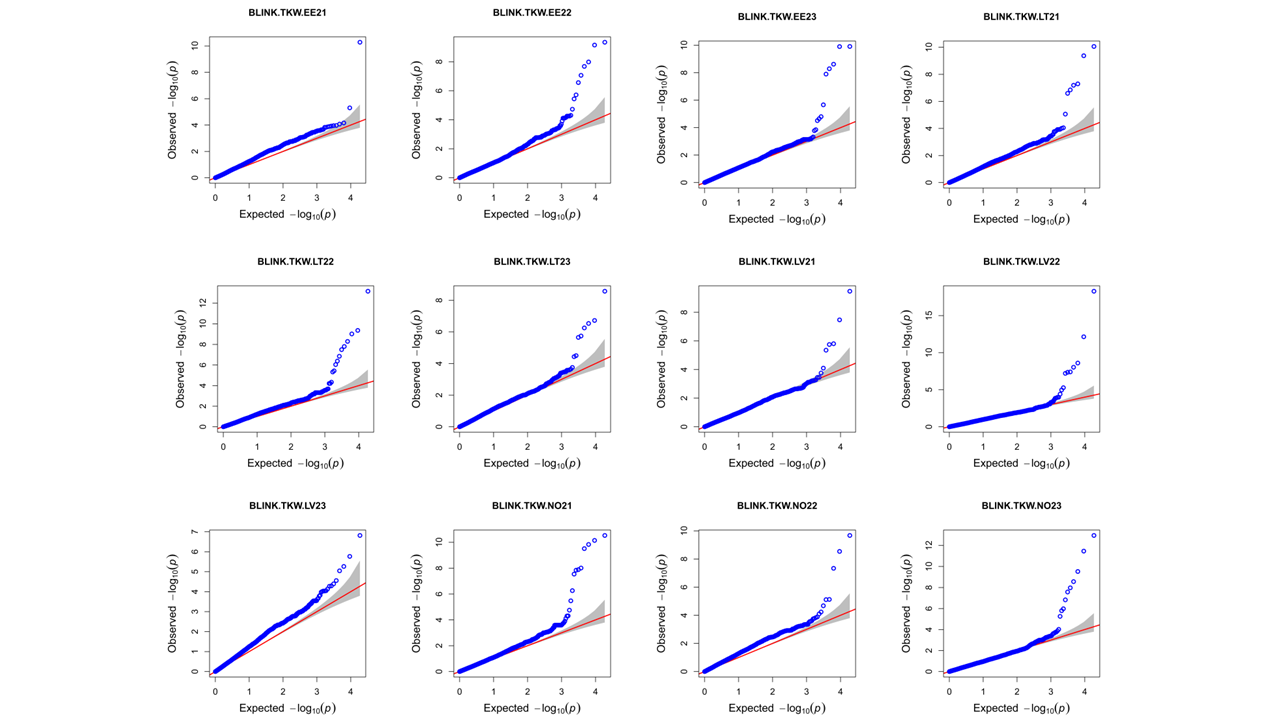


**Figure S27.** Q-Q plots for thousand kernel weight (TKW).


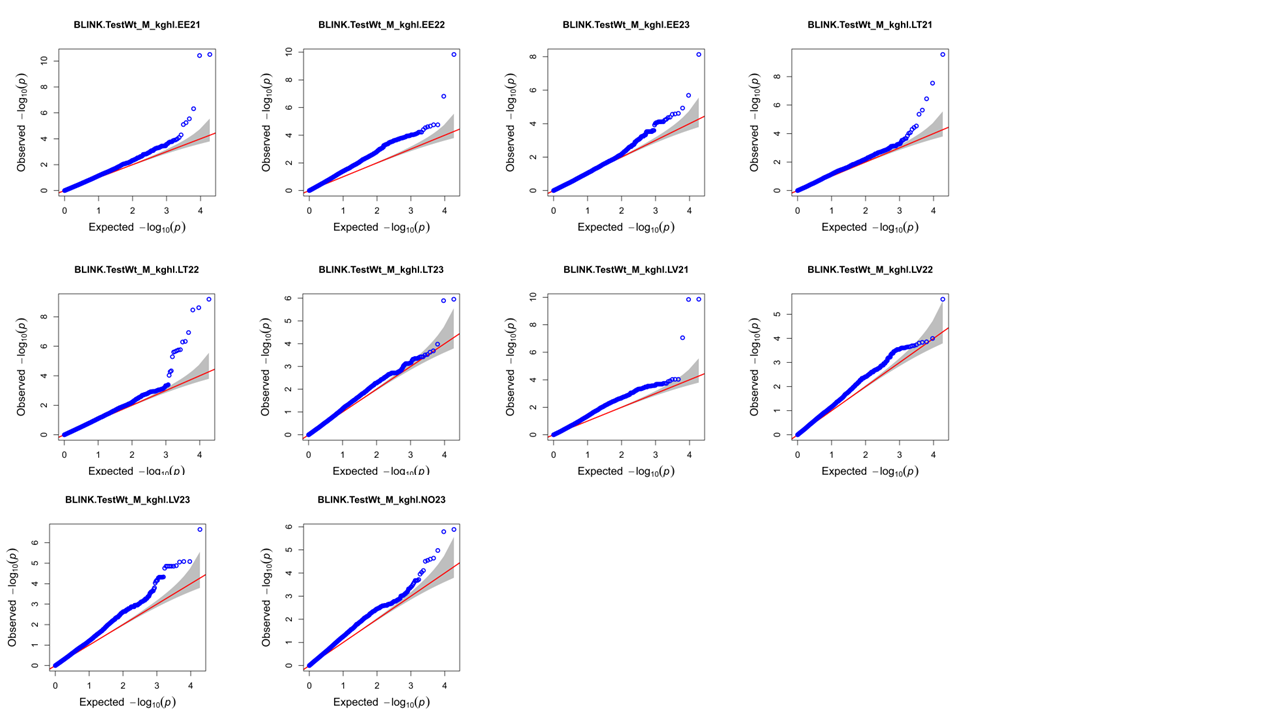


**Figure S28**. Q-Q plots for test weight (TW).


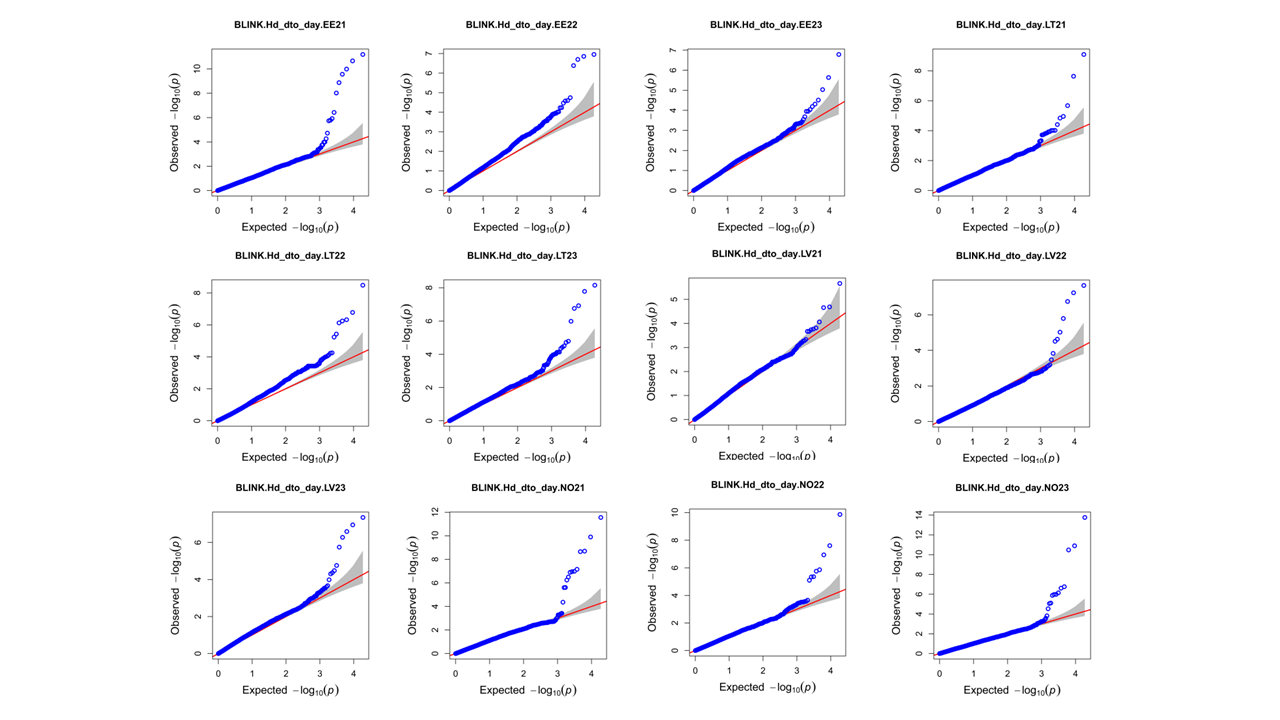


**Figure S29**. Q-Q plots for days to heading (DH).


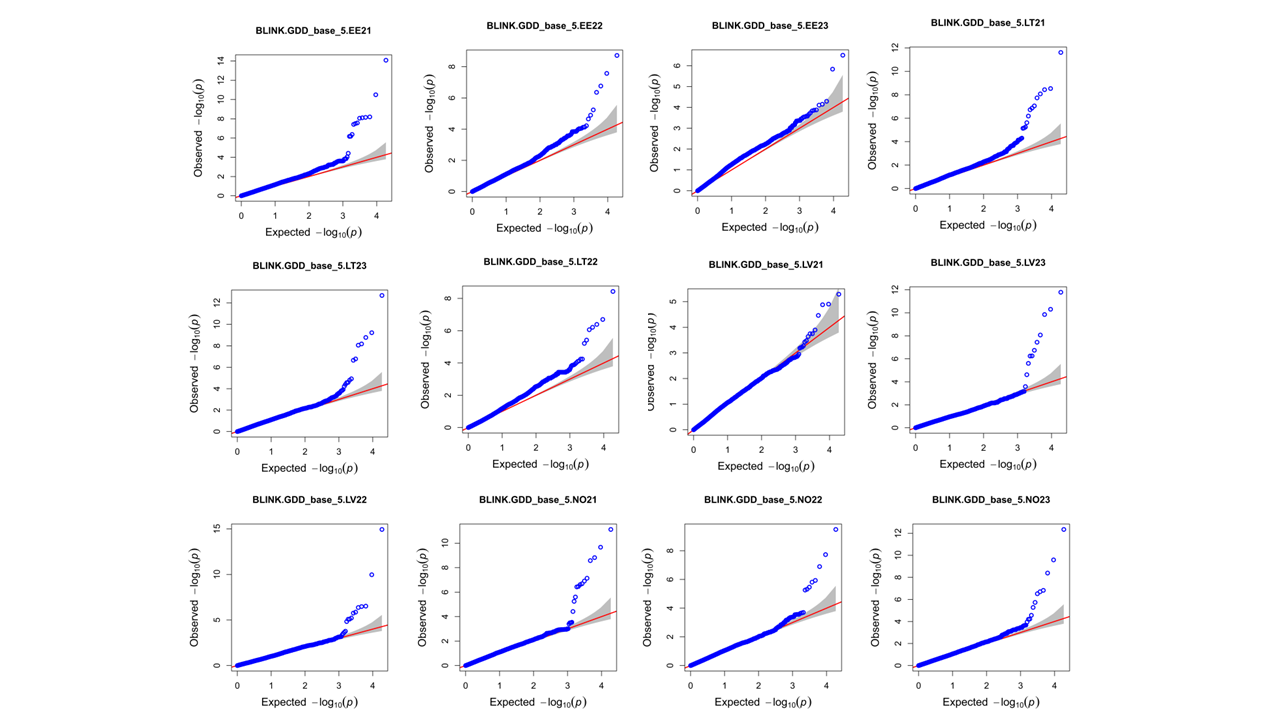


**Figure S30**. Q-Q plots for growing degree days to heading (GDD).


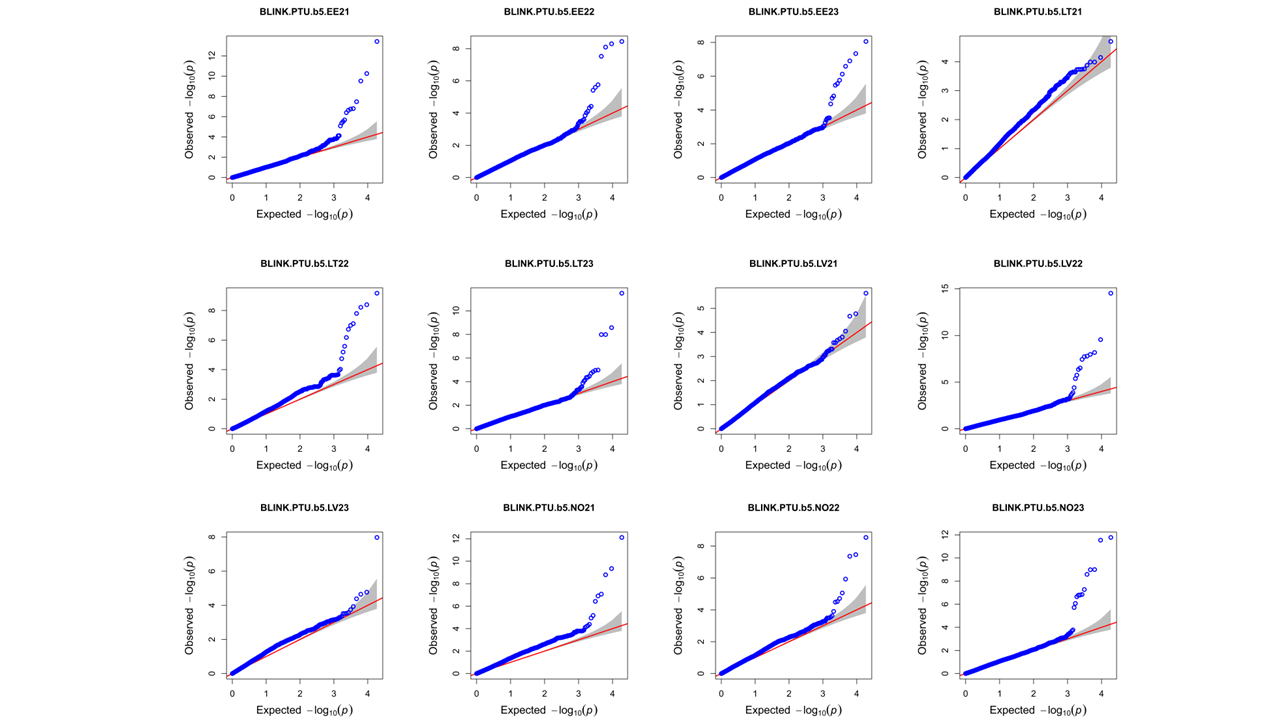


**Figure S31**. Q-Q plots for photothermal units to heading (PTU).


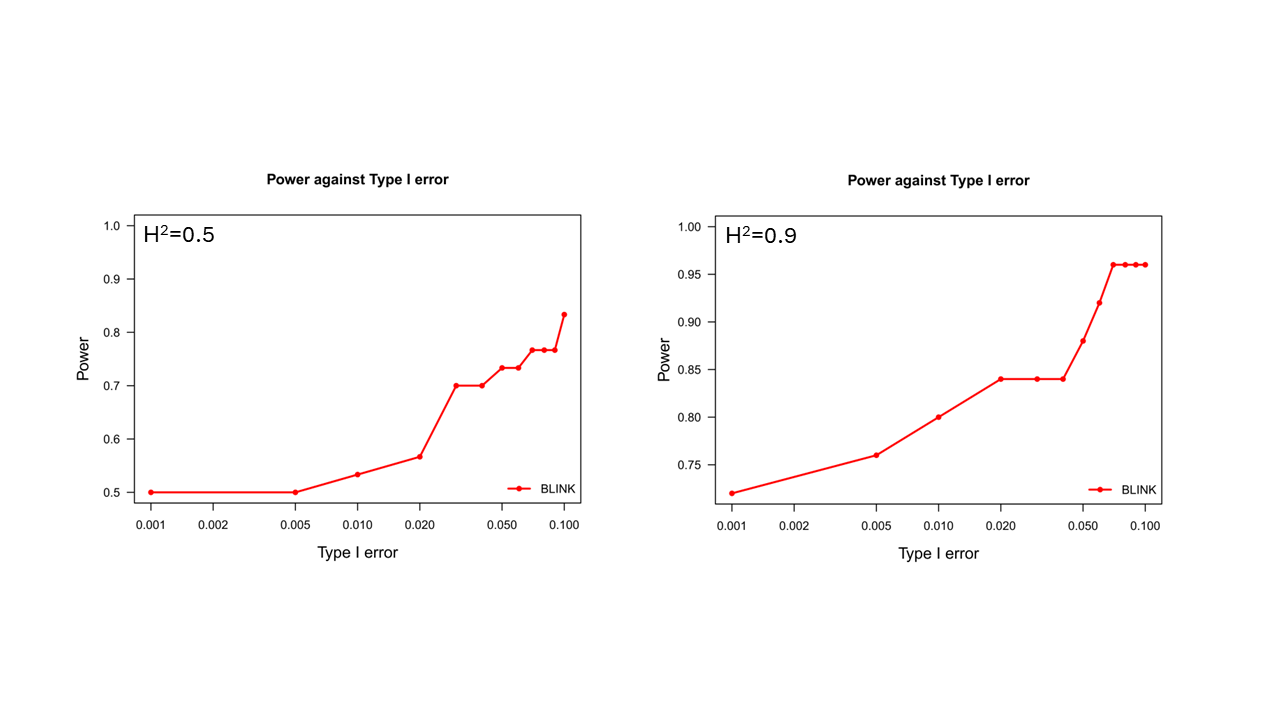


**Figure S32**. GWAS statistical power against Type I error for BLINK method in two scenarios: moderate (H^2^=0.5) and high (H^2^=0.9) broad-sense heritability.


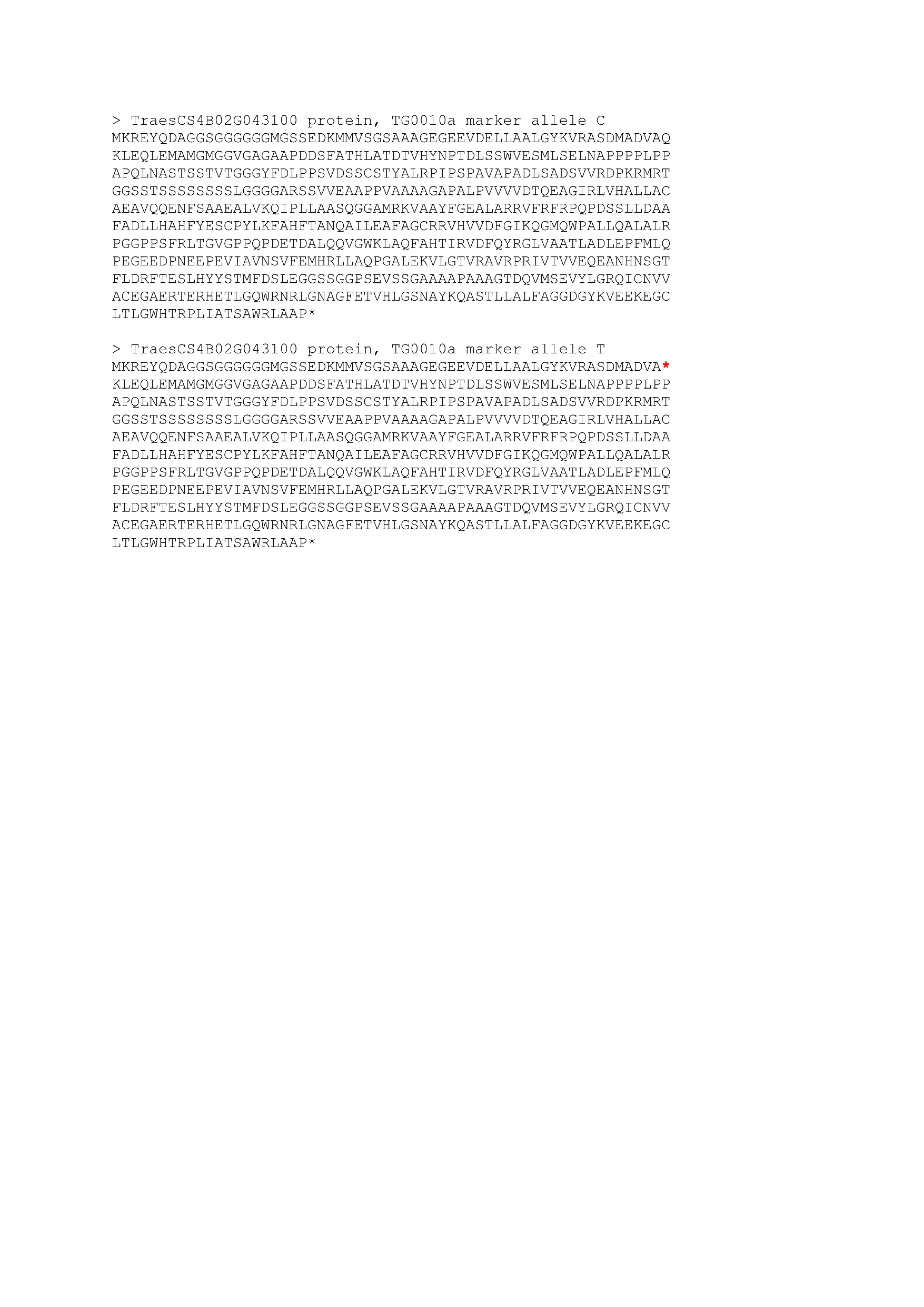


**Figure S33**. TG0010a marker allele substitution effect on TraesCS4B02G043100 protein (DELLA protein Rht-B1). The nucleotide substitution at TG0010a site from C -> T leads to the introduction of premature stop codon (*) Q60Stop.

**
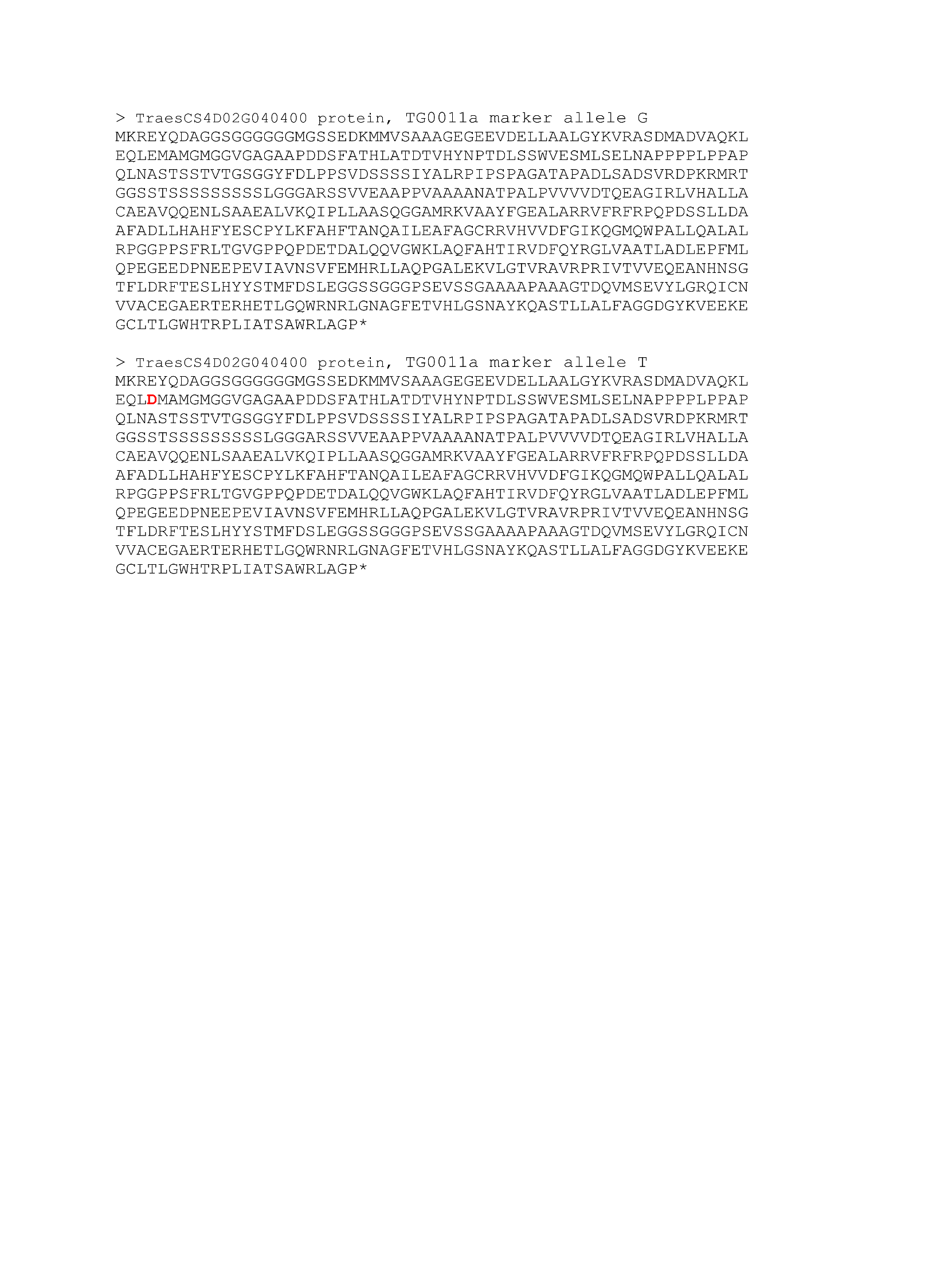
**

**Figure S34.** TG0010a marker allele substitution effect on TraesCS4D02G040400 protein (DELLA protein Rht-D1). SNP variation at the site from G -> T leads to amino acid Glu -> Asp (E64D) substitution in the encoded protein.


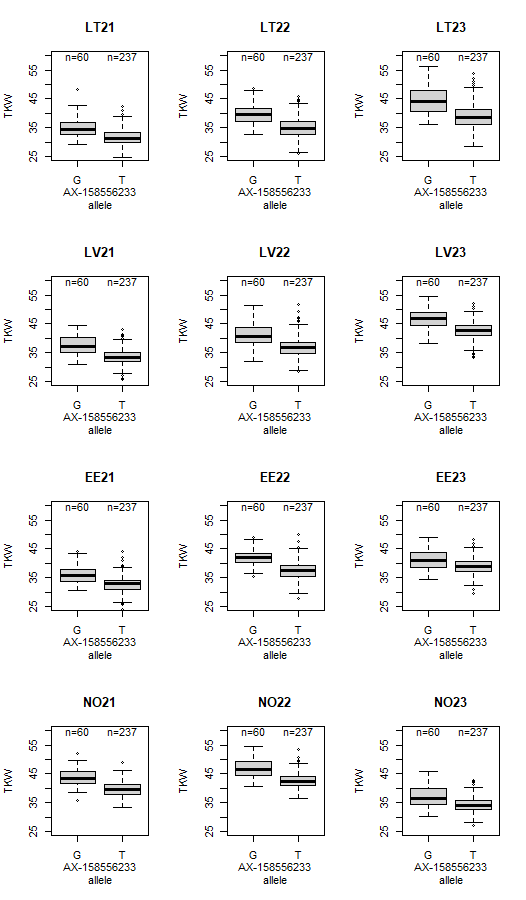


**Figure S35**. The effect of allelic variation of AX-158556233 marker on thousand kernel weight (TKW) in different trials.


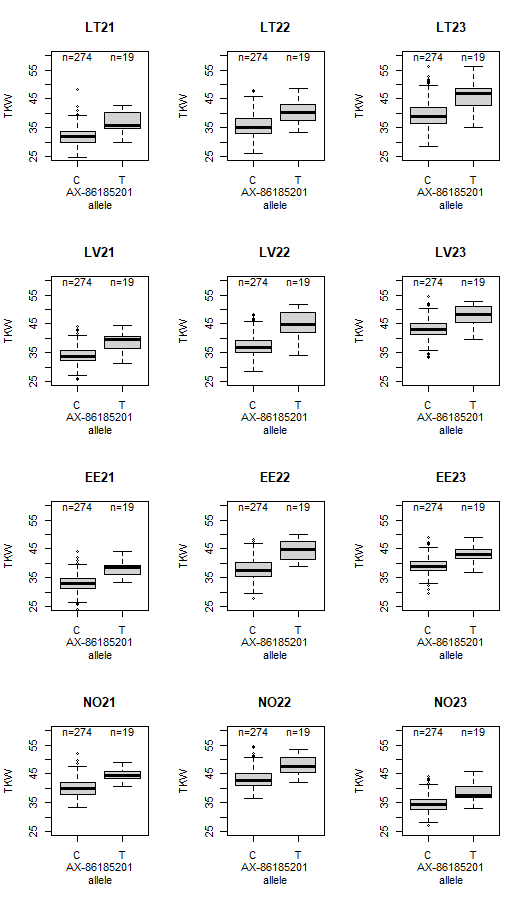


**Figure S36**. The effect of allelic variation of AX-86185201 marker on thousand kernel weight (TKW) in different trials.
